# Biota monitoring and the Water Framework Directive—can normalization overcome shortcomings in sampling strategies?

**DOI:** 10.1007/s11356-016-7442-2

**Published:** 2016-08-18

**Authors:** Annette Fliedner, Heinz Rüdel, Diana Teubner, Georgia Buchmeier, Jaqueline Lowis, Christiane Heiss, Jörg Wellmitz, Jan Koschorreck

**Affiliations:** 1 Fraunhofer Institute for Molecular Biology and Applied Ecology (Fraunhofer IME), 57392 Schmallenberg, Germany; 2 Department of Biogeography / ESB, Trier University, 54286 Trier, Germany; 3 Bavarian Environmental Agency (LfU), Demollstr. 31, 82407 Wielenbach, Germany; 4 North Rhine-Westphalian State Agency for Nature, Environment and Consumer Protection (LANUV), Leibnizstraße 10, 45659 Recklinghausen, Germany; 5 German Environment Agency (Umweltbundesamt), 06813 Dessau-Rosslau, Germany

**Keywords:** Biota monitoring, Water Framework Directive, Normalization, HCB, Hg, PFOS, Environmental Specimen Bank

## Abstract

We compare the results of different monitoring programs regarding spatial and temporal trends of priority hazardous substances of the European Water Framework Directive (WFD). Fish monitoring data for hexachlorobenzene (HCB), mercury (Hg), and perfluorooctane sulfonic acid (PFOS) sampled in German freshwaters between the mid-1990s and 2014 were evaluated according to the recommendations of the 2014 adopted WFD guidance document on biota monitoring, i.e., normalization to 5 % lipid content (HCB) or 26 % dry mass (Hg, PFOS) and adjustment to trophic level (TL) 4. Data of the German Environmental Specimen Bank (ESB) (annual pooled samples of bream) were compared to monitoring data of the German federal states (FS), which refer to individual fish of different species. Significant decreasing trends (*p* < 0.01) were detected for Hg in bream (*Abramis brama*) sampled by both, the ESB and the FS between 1993 and 2013 but not for FS samples comprising different fish species. Data for HCB and PFOS were more heterogeneous due to a smaller database and gave no consistent results. Obviously, normalization could not compensate differences in sampling strategies. The results suggest that the data treatment procedure proposed in the guidance document has shortcomings and emphasize the importance of highly standardized sampling programs in trend monitoring or whenever results between sites have to be compared.

**Electronic supplementary material:**

The online version of this article (doi:10.1007/s11356-016-7442-2) contains supplementary material, which is available to authorized users.

## Introduction

The European Water Framework Directive (WFD 2000/60/EC; EC 2000) stipulates systematic monitoring to evaluate the chemical quality of fresh and estuarine waters. In this context, priority substances have been identified on a European level together with corresponding environmental quality standards (EQS). These EQS serve as benchmark for potential risks, indicating chemical quality of water bodies and directing need for future action (e.g., emission control measures).

Recent changes in legislation specified monitoring obligations in biota for 11 priority substances. Respective EQS_Biota_ were derived (Directive 2008/105/EC (EC 2008); 2013/39/EU (EC 2013)) which address two protection goals: (a) protection of human health against risk from freshwater food and (b) protection of top predators from secondary poisoning. Nine of these substances shall be monitored in fish, i.e., polybrominated diphenyls (PBDE), hexachlorobenzene (HCB), hexachlorobutadiene (HCBD), mercury and its compounds (Hg), Dicofol, perfluorooctane sulfonic acid (PFOS), dioxins, furans and dioxin-like PCB (PCDD/F + dl-PCB), hexabromocyclododecane (HBCDD), and heptachlor and heptachlor epoxide.

In 2014, the new WFD Guidance Document No. 32 on biota monitoring (EC 2014) was published which aims at the harmonization of biota monitoring with respect to selection of species and type of samples (e.g., tissue or whole fish, pool or individual, sample size, sampling frequency). The document also includes recommendations for data handling with the intention to facilitate the comparison between data from different waters and member states and harmonize the compliance check with the EQS values.

In Germany, fish monitoring has a history of more than 30 years, providing a valuable backbone for the analysis of long-term trends. However, depending on the aim of the monitoring (i.e., human health or environmental protection), different sampling strategies were applied which hinders the direct comparison of data from different programs. In addition to the monitoring programs of the German federal states (FS), the Environmental Specimen Bank (ESB) established a highly standardized monitoring system in the early 1990s, which now extends over the three major stream systems in Germany, namely Rhine, Elbe, and Danube and some tributaries (German Environment Agency 2016).

With the help of the data handling recommendations in the new guidance document (EC 2014) (i.e., normalization to lipid content, respectively, dry mass as well as adjustment to a common trophic level (TL)), it now appears feasible to evaluate and compare the existing monitoring data of the FS and the ESB. Ideally, the data can be linked to newly generated ones and support the design of future monitoring programs.

In the present study, the proposed data treatment procedure was applied to fish monitoring data of the three priority substances HCB, Hg, and PFOS. These substances were chosen because they represent different modes of accumulation.

HCB is a lipophilic compound and accumulates mainly in lipid-rich tissue of organisms. It enriches in the food chain (Moermond and Verbruggen 2013) and thus poses a threat to human consumers and fish-eating top predators. Based on studies in 17 North American lakes, Houde et al. (2008) derived an average trophic magnification factor (TMF) of 2.9 that is also supported by an extensive literature study by Moermond and Verbruggen (2013).

Organic and inorganic Hg binds to sulfhydryl (SH) groups within proteins and high concentrations are found, e.g., in muscle tissue of fish. In aquatic ecosystems, Hg and especially its organic form methyl-Hg have a high potential for biomagnification. Lavoie et al. (2013) derived a mean TMF for total Hg of 4.3 ± 4.8 based on a meta-analysis of studies on 101 freshwater food webs worldwide. The trophic transfer of mercury, however, strongly depends on the characteristics of the geochemical cycle in the respective water body including pH, DOC, and productivity (Åkerblom et al. 2014; Clayden et al. 2013; Lavoie et al. 2013).

PFOS binds to proteins and accumulates mainly in protein-rich tissue like the liver, kidney, and blood (Ahrens et al. 2009; Kelly et al. 2009). Like HCB and Hg, PFOS is very persistent with a high potential for bioaccumulation and biomagnification. A wide range of TMFs (1.4–19.6) have been reported depending on the studied ecosystems and food chains (Houde et al. 2011). Based on an extensive food web study of Lake Taihu in China, Fang et al. (2014) calculated a mean TMF of 3.6.

HCB and Hg have been monitored for many years, and the existing database is large. In contrast, PFOS can be considered as a relatively new substance in environmental monitoring because it was only in 2001 that practical analytical methods became available which allowed its detection in environmental samples (Giesy and Kannan 2001; Hansen et al. 2001). Accordingly, much less data are available from the FS monitoring, whereas archived ESB samples allowed a retrospective analysis of PFOS back to 1995.

The aims of this study are (1) to evaluate and compare the existing monitoring data of the FS and ESB based on normalized data following the EU approach under the WFD, (2) to assess whether data treatment can substitute a standardized sampling strategy in trend monitoring by comparing trends from existent FS data with those from the ESB, and (3) to evaluate whether existing monitoring data can be used for a (retrospective) compliance checking.

## Materials and methods

### Data compilation

The ESB data are partly available on the ESB Web site (www.umweltprobenbank.de/en). Additional data were retrieved from ESB reports and publications (e.g., Theobald et al. 2011).

The ESB samples bream (*Abramis brama*) annually since 1993. Fish are collected at 16 riverine sampling sites located in the rivers Rhine, Elbe, Danube, Saar, Saale, and Mulde and one lake site (Lake Belau in Northern Germany). Sampling and processing are highly standardized following standard operating procedures (German Environment Agency 2016; Klein et al. 2012; Paulus et al. 1996; Rüdel et al. 2008). Per site and year, the filets of approximately 20 8–12-year-old bream are pooled and cryo-homogenized to annual samples. After chemical characterization, subsamples of the pool samples are archived at a temperature below −150 °C in the inert atmosphere above liquid nitrogen. Chemical analysis of HCB and PFOS in the ESB samples was performed by Eurofins GfA Lab Service GmbH, Hamburg, Germany. The analysis of HCB was performed using a HRGC/HRMS analog protocol to a method described by Lohmann et al. (2014), which includes the use of ^13^C-labeled internal standards, column extraction by means of hexane/acetone, and a multicolumn clean-up procedure including alumina and florisil. PFOS was analyzed by liquid chromatography and tandem mass spectrometry (LC/MS-MS) using ^13^C-labeled internal standards after ultrasonic extraction with appropriate polar solvents (e.g., methanol) and clean-up by applying carbon black. The lipid content was determined gravimetrically in the tissue extracts. Since 2000, Hg has been analyzed at Fraunhofer IME by dedicated atomic absorption spectrometry (AAS) methods applying direct mercury analyzer (DMA) instruments (Rüdel et al. 2010). The laboratories hold accreditations for the applied methods, and all respective quality assurance/quality control (QA/QC) requirements were met. Precision and accuracy were checked by analyzing in-house QC-pool samples, sample material of previous interlaboratory proficiency studies, and/or certified reference materials.

Data from the federal states monitoring stem from publications and the Elbe Data Information System FIS of the River Basin Community Elbe (http://www.fgg-elbe.de/elbe-datenportal.html). Furthermore, comprehensive data sets of the federal states fish monitoring were kindly provided by the following institutions (in alphabetical order): Bavarian Environmental Agency, Lower Saxony Water Management, Coastal Defence and Nature Conservation Agency, Mecklenburg-Western Pomeranian Agency for the Environment, Nature Conservation and Geology, North Rhine-Westphalian State Agency for Nature, Environment and Consumer Protection, Saxony-Anhalt State Office for Consumer Protection, Saxon State Office for the Environment, Agriculture and Geology, and Thuringian Regional Office for the Environment and Geology. Chemical analysis of samples from early monitoring periods was performed by FS laboratories accredited according to national guidelines. In recent years, the EU Directive 2009/90/EC (EC 2009) on technical specifications for chemical analysis and monitoring of water status has been implemented.

Trophic levels of fish and fish dry weight data were retrieved from FishBase (Froese and Pauly 2016) and are summarized in Table S1 (Online Resource). Trophic magnification factors (TMFs) used in the normalization procedure come from publications of Houde et al. (2008), Lavoie et al. (2013), and Fang et al. (2014).

### Data normalization

HCB concentrations of fish of different species and sizes as well as different water bodies and years were normalized to 5 % lipid content and adjusted to a trophic level of 4.0 (TL 4) according to the new WFD guidance document on biota monitoring (EC 2014). Lipid normalization based solely on measured lipid contents of ESB and FS samples. Correlations between HCB and lipid concentrations were checked by calculating Pearson’s correlation coefficient using Microsoft Excel (Version 2010). Hg and PFOS concentrations were normalized to 26 % dry mass before adjustment to TL 4 (EC 2014). ESB dry mass normalization used measured dry mass data while normalization of the FS data based on default dry mass values derived from FishBase (Froese and Pauly 2016). A default value of 0.25 was applied in the case of missing data. Normalization was performed as follows (EC 2014):

1 $$ \begin{array}{c}\hfill Con{c}_{TL\  adjust. norm}= Con{c}_{meas}\times TM{F}^{\left(4.0- TL(x)\right)}\times \frac{0.05}{lipid\ (x)}\left(for\ HCB\right)\hfill \\ {}\hfill \left(\mathrm{respectively}\times \frac{0.26}{dry\  mass\ (x)}\ \left(for\ Hg\ \mathrm{and}\ \mathrm{PFOS}\right)\right)\hfill \end{array} $$

Conc_TL-adj.norm_: concentrations normalized to dry mass or lipid and adjusted to TL 4.0;
Conc_meas_: measured concentration in mg/kg ww;
TMF: trophic magnification factor (i.e., 2.9 for HCB; 4.3 for Hg; 3.6 for PFOS; references are given in the section “Data compilation”)
TL(x): trophic level of the respective fish species x retrieved from FishBase;
lipid (x): measured lipid concentration of sample x;
dry mass (x): dry mass of fish species x retrieved from FishBase (for FS samples), respectively, measured dry mass of sample (for ESB samples).

### Verification of default TL for bream

The actual trophic levels of bream were calculated for five ESB sites based on the ^15^N/^14^N ratios of fish and suspended particulate matter. Cryo-milled muscle tissue samples were freeze-dried and analyzed raw. Baselines for δ^15^N values, i.e., trophic level (TL) = 1.0, refer to organic matter of annually composed samples of suspended particulate matter (SPM). Sampling of SPM was performed by the Institute for Hydrogeology and Environmental Geochemistry, Freie Universität Berlin, Germany, based on protocols by Schulze et al. (2007). It is assumed that the δ^15^N values of SPM resemble those of primary producers because the organic fraction of SPM is mainly composed of plant litter, phytoplankton, macrophytes, and detritus (Dalu et al. 2016; Hope et al. 1994; Kohzu et al. 2009; compositions may vary depending on local sources/influences and sampling techniques). Furthermore, SPM represents a potential food sources for primary consumers. Data of fish and SPM from identical sampling sites and overlapping sampling periods were used for calculating the TLs for bream samples.

Analysis was performed using an elemental analyzer (Flash EA 1112, Thermo Scientific, Milan, Italy) coupled to an isotope ratio mass spectrometer (Delta V Advantage with a Conflo IV interface, Thermo Scientific, Bremen, Germany) at LIENSs stable isotope facility of the University of La Rochelle, France. Results are expressed in the δ unit notation (see, e.g., Post 2002) as deviations from a standard (N_2_ in air):

2 $$ {\delta}^{15}\mathrm{N}=\left[\frac{R(sample)}{R\ (standard)}-1\right]\times {10}^3\mathrm{withR}={}^{15}\mathrm{N}/{}^{14}\mathrm{N} $$

Reference gas was calibrated against reference materials (IAEA-N2, IAEA-NO-3, IAEA-600). The analytical precision, based on the analyses of acetanilide (Thermo Scientific) used as laboratory internal standards interspersed among the samples, was <0.15 ‰ for δ^15^N values.

Trophic levels of the bream were calculated as follows (McCutchan et al. 2003; Post 2002):

3 $$ TL=\frac{\delta^{15}N(fish)\left[{\mbox{\fontencoding{U}\fontfamily{wasy}\selectfont\char104}} \right]-{\delta}^{15}N(foodsource)\left[{\mbox{\fontencoding{U}\fontfamily{wasy}\selectfont\char104}} \right]}{3.4}+1 $$

δ^15^N_food source_ δ^15^N value of the organic matter of the SPM sampled at the respective fish sampling site.

### Data analysis

Time trends for HCB, Hg, and PFOS were statistically analyzed using a software tool from the German Environment Agency (Umweltbundesamt (UBA); LOESS-Trend, Version 1.1, based on Microsoft Excel). This tool fits a locally weighted scatterplot smoother (LOESS; fixed window width of 7 years) through the yearly contaminant levels and then tests for significance of linear and non-linear trend components by means of an analysis of variance (ANOVA) following the approach of Fryer and Nicholson (1999). Trend analysis was based on the antilog of the mean of log-transformed concentrations after normalization according to the Guidance Document No. 32 (EC 2014).

To evaluate in how far data treatment can substitute standardized sampling, the complexity of the analyzed data was reduced step-by-step by analyzing trends at three different levels. At each level, ESB data were compared to data from the federal states: The first level included all data from German freshwaters, i.e., annual pooled ESB samples, respectively, individual fish data from the FS from all sampling sites, irrespective of the water body. The second level focused on only one of the major German streams (Elbe, Danube, and Rhine) depending on the available FS data. The third level concentrated on one sampling site in the respective stream. If the FS database was too poor, the data of sampling sites upstream and close to the respective ESB site were combined. At each level, the analysis of FS data was performed twofold, for (1) samples comprising all fish species and (2) samples from only one fish species. If not enough data for one single fish species were available, the data of fish of similar trophic levels as bream were analyzed together (TL 2.0–3.2).

The stepwise comparison of ESB and FS data was used to test the hypothesis that increasing similarity between the data from the two monitoring concepts will increase the similarity of the temporal fish data.

## Results and discussion

### Database

In total, more than 15,000 data sets were analyzed (Table 1). The majority of the FS data refer to Hg and HCB, which have been monitored for a long time now and are included, e.g., in the FIS database. FS monitoring of PFOS started only in 2005. The ESB data go back to the mid-1990s making use of retrospective trend analysis of archived samples.

**Table 1:** Summary of the compiled fish monitoring data for hexachlorobenzene (HCB), mercury (Hg), and perfluorooctane sulfonic acid (PFOS) from German freshwaters. ESB data refer to pooled muscle samples of about 20 fish each; FS samples refer to muscle samples of individual fish

Substance	CAS No	EQS_Biota_ μg/kg *w*/*w*	ESB		Federal States and contacted institutions	
			*N*	covered period	*N*	first year/last year
Hexachlorobenzene (HCB)	118-74-1	10	296	1993–2013	6655	1980 / 2013
Mercury and its compounds (Hg)	7439-97-6	20	296	1993–2013	7001	1978 / 2013
Perfluorooctane sulfonic acid/sulfonate (PFOS)	1763-23-1	9.1	180	1995–2014	1268	2005 / 2013

### Species distribution

ESB sampling is highly standardized and focuses entirely on bream (*A. brama*). The publicly available FS data of the FIS database and the additionally provided data of the federal states refer to about 35 different fish species. The most commonly sampled non-predatory fish were bream (*A. brama*), chub (*Squalius cephalus)*, roach (*Rutilus rutlius*), and eel (*Anguilla anguilla*), while pikeperch (*Sander lucioperca*), pike (*Esox lucius*), and perch (*Perca fluviatilis*) were the most frequently sampled predatory fish. The fish species distribution reflects the monitoring objective of the different federal states as well as the abundance of the species in the respective river systems.

Based on the trophic levels given in FishBase, about 50 % of the FS data for Hg refer to species that occupy the trophic levels 2.0–3.2 (HCB: 48 %, PFOS: 35 %), about 31 % to fish of trophic levels 3.3–3.9 (HCB: 33 %, PFOS: 44 %), and about 19 % are predatory fish of TL ≥ 4.0 (HCB: 19 %, PFOS: 24 %). The trophic levels of the most commonly monitored fish species are summarized in Table S1 (Online Resource).

For five ESB sites, the actual trophic levels of bream were calculated based on stable isotope analysis of nitrogen. According to these data, bream occupied trophic levels of 2.8–3.5 (mean 3.1, Online Resource, Table S2) which is in good agreement with the TL value of 3.1 given in FishBase and used in the normalization procedure.

### Biometrical data

Weight, length, age, sex, dry mass, and lipid content are included in all ESB data sets. Less biometrical data were available for the FS data: Depending on substance, 86–99 % of the data sets included fish weights and 52–97 % fish length. Age data was available only in 6–23 % cases and data on sex in 0–48 %. Lipid content was included in 21 % of the HCB data sets. No dry mass data was available for FS samples.

### Data analysis

Analysis of the trend data was performed for overlapping sampling periods of the ESB and FS monitoring programs (Table 2). An additional analysis included all available data (i.e., also those years for which only ESB or FS data were available). The respective statistical parameters and the ESB data sorted by sampling sites including trends are compiled in the Online Resource (Tables S3–S14).

**Table 2:** HCB, Hg, and PFOS concentrations (μg/kg wet weight) in muscle tissue of fish from German freshwaters sampled under the Environmental Specimen Bank program (ESB) and under the fish monitoring programs of the federal states (FS). Data of the ESB refer to pooled annual samples of approximately 20 bream each while FS data refer to individual fish of different species. Left columns: concentrations adjusted to a standard fish of 5 % lipid content (HCB), respectively, 26 % dry mass (Hg, PFOS) and trophic level 4 (TL 4). Right, shaded columns: originally reported concentrations

		ESB		FS	
HCB [μg/kg *w*/*w*]	first year/last year	2000/2010		2000/2010	
	N_waterbodies_	7		49	
	N_sampling sites_	17		103	
	N_samples_	168		1393	
		normalized: 5 % lipid; TL 4	reported	normalized: 5 % lipid; TL 4	reported
	Mean	67	15	13	23
	±SD	81	14	30	55
	Median	29	11	6	8
	Min	1.4	<0.2	<0.1	<0.2
	Max	416	77	591	1331
Hg [μg/kg *w*/*w*]	first year/last year	1993/2013		1993/2013	
	N_waterbodies_	7		90	
	N_sampling sites_	17		204	
	N_samples_	296		6417	
		normalized: 26 % DM, TL 4	reported	normalized: 26 % DM, TL 4	reported
	Mean	999	239	858	357
	±SD	595	144	917	381
	Median	904	214	562	258
	Min	102	21	<40	<10
	Max	3987	881	10,602	9080
PFOS [μg/kg *w*/*w*]	first year/last year	2005/2010		2005/2010	
	N_waterbodies_	7		64	
	N_sampling sites_	17		140	
	N_samples_	68		1268	
		normalized: 26 % DM, TL 4	reported	normalized: 26 % DM, TL 4	reported
	Mean	92	25	133	67
	±SD	56	16	342	164
	Median	78	20	38	22
	Min	2.3	0.6	<0.8	<0.2
	Max	234	70	4852	2749

*DM* dry mass

#### Hexachlorobenzene

HCB was analyzed in ESB samples from the years 1993–2013 (Online Resource, Table S3).

The data of the FS monitoring of HCB go back to the 1980s. Lipid contents, however, were available only for 21 % (*n* = 1393) of the data sets and refer to the years 2000–2010. Correlation analysis for these data revealed a strong relationship between HCB and lipid contents (Pearson’s correlation coefficient, *p* < 0.0001).

In the years 2000–2010, HCB levels in ESB samples range between <0.2 and 77 μg/kg *w*/*w* (corresponding to 1.4–416 μg/kg *w*/*w* when adjusted to a standard fish of 5 % lipid content and TL 4). Highest levels were detected at the ESB sites in the Elbe and Mulde while levels in bream from the Danube were generally low.

In the FS samples, reported HCB concentrations ranged between <0.2 and 1331 μg/kg *w*/*w* and between <0.1 and 591 μg/kg *w*/*w* when normalized to 5 % lipid content and TL 4 (Table 2, details in Online Resource, Table S4).

Normalization resulted in higher HCB concentrations in the ESB samples but lower values in the FS samples. Since HCB accumulates in fatty tissue, the lipid content of the fish is a crucial factor. Of all ESB samples, more than 50 % had lipid contents below the fat standard of 5 % recommended in the EU guidance document. For these samples, the normalization resulted in higher HCB concentrations. In contrast, 68 % of the fish analyzed by the FS had lipid contents higher than 5 % (most of them were eel), and normalization resulted in lower HCB levels. Furthermore, the majority (77 %) of the FS fish belonged to higher trophic levels than bream, so that the adjustment to TL 4 had stronger effects on the ESB data.

Based on normalized data, the EQS_Biota_ for HCB of 10 μg/kg *w*/*w* was exceeded at 10 of the 17 ESB sampling sites in 2013 (or 2012 at the sampling site Weil/Rhine; Online Resource, Table S3). Regarding the FS data and the years 2009 and 2010, six of 26 FS sites exceeded the EQS_Biota_ (in the lower Rhine “Düsseldorf to Meerbusch,” “Rees to Grietherort,” “Aalschokker at Grietherort,” and “Emmerich,” as well as the sites “Elbe Abstiegskanal” and “Haiming” in the river Salzach).

**Fig. 1:**
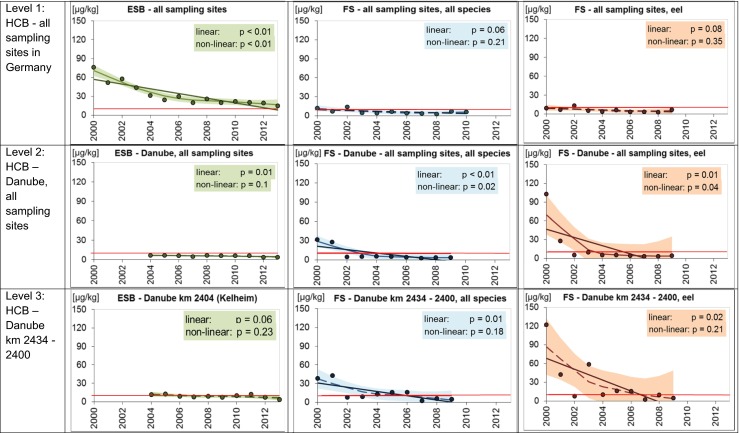
Temporal trends in HCB concentrations (μg/kg; wet weight-based) in muscle tissue of freshwater fish in Germany sampled by the German Environmental Specimen Bank (ESB) and the federal states (FS). Data are adjusted to a standard fish of trophic level 4.0 and 5 % lipid content. Shown are the antilog data of annual means of log-transformed concentrations after normalization. The *lines* represent the results of the linear regression (*solid* for significant linear trend, *dashed* for not significant) and the LOESS smoother (*solid* for significant non-linear trend, *dashed* for not significant).The *shaded areas* mark the 95 % confidence intervals of the LOESS function. Data basis: ESB: annual pool samples of approximately 20 bream each; FS: individual fish of different species. *Red line* EQS_Biota_ (10 μg/kg wet weight)

Figure 1 summarizes the results of the trend analysis for HCB at different levels of complexity.

For the FS data, trend analysis was performed for the whole data set (including all species) as well as for eel as representative of a single fish species. Eel was chosen because most of the HCB data sets that included lipid contents referred to this species (e.g., for the period 2000–2009, 20–100 % depending on year; Online Resource, Tables S4, S5, S6) while considerably less lipid data were available for bream and fish of trophic levels TL 2.0–3.2.

HCB decreased significantly in bream sampled by the ESB between 2000 and 2013 (linear trend, *p* < 0.01) when all available data from this monitoring program were included in the analysis (Fig. 1, level 1). In contrast, no significant trends were detected for fish sampled by the FS in German freshwaters between 2000 and 2009/2010 (linear trends, all species: *p* = 0.06; eel: *p* = 0.08).

In view of the available FS data for HCB, further trend analysis at the next steps (levels 2 and 3) focused on the river Danube. This, however, had the drawback that ESB data were available only since 2004.

When considering the ESB data from all Danube sampling sites together at level 2 (Fig. 1), a significant decreasing trend was detected for the period 2004–2013 (linear trend, *p* = 0.01). Significantly decreasing HCB concentrations were also detected in fish sampled by the FS in the Danube between 2000 and 2009 (linear trends, all species: *p* < 0.01, eel: *p* = 0.01). When trend analysis included only the common sampling years 2004–2009, neither ESB nor FS data showed significant trends.

At level 3, trend analysis had to rely on relatively small sample numbers: for the common sampling period 2004–2009, six annual pool samples were available from the ESB site Kelheim (km 2404) and 24 individual fish (all species), respectively, 14 individual eel from the close-by FS sites between Danube km 2434 and 2400.

Nevertheless, slightly decreasing HCB concentrations were noticeable for both ESB and FS time series, but trends were not significant. When extending the trend analysis for the ESB data to the year 2013 (Fig. 1, level 3), the trend became more pronounced but was still not significant (linear trend, *p* = 0.06). In the case of the FS data, inclusion of earlier years (2000–2009) resulted in significant decreasing trends (linear trends, *p* = 0.01 (all species, *n* = 41) and *p* = 0.02 (eel, *n* = 23)).

Taken together, the findings indicate that normalization to 5 % lipid content and TL 4 did not overcome differences between individual fish or species at any of the three levels. Reducing complexity by focusing on one species only did not result in more homogenous data.

The most likely reason for this is the high variability within the eel data. HCB levels differed considerably between individual eels sampled in one year at the same or close-by sites. For example, for HCB and eel sampled between Danube km 2434 and 2400, standard deviations of the normalized data ranged between 41 and 128 %, depending on the year. This can be explained, on the one hand, by the opportunistic feeding strategy of eel, which includes fish, amphipods, decapod crustaceans, and terrestrial species (Froese and Pauly 2016; Jacoby and Gollock 2014). On the other hand, the duration of exposure may have varied (which could not be analyzed due to lack of fish age data).

Both factors complicate the interpretation of trends based on eel data and question the use of eel in chemical monitoring under the WFD.

#### Mercury

Between 1993 and 2013, reported Hg concentrations ranged between 21 and 881 μg/kg *w*/*w* in the ESB samples (Table 2, Online Resource, Table S7). The concentration range was wider in fish sampled by the FS (i.e., reported concentrations of <10–9080 μg/kg *w*/*w*, Online Resource, Table S8). This is mainly because the FS data refer to individual fish and a large variety of different fish species and sizes whereas the ESB data refer to pool samples of bream only. In 2013, the EQS_Biota_ for Hg of 20 μg/kg *w*/*w* was exceeded at all ESB and FS sites.

Normalization to 26 % dry mass and TL 4 led to higher Hg concentrations in both data sets because the majority of the sampled fish (ESB: 100 %, FS: 81 %) belong to trophic levels below 4.0. Adjustment to TL 4—especially when using a high TMF of 4.3 as done here—therefore results in higher Hg levels.

**Fig. 2:**
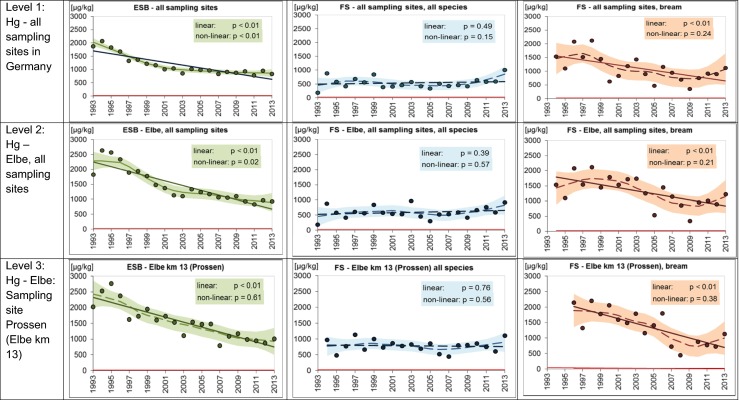
Temporal trends in Hg concentrations (μg/kg; wet weight-based) in muscle tissue of freshwater fish in Germany sampled by the German Environmental Specimen Bank (ESB) and the federal states (FS). Data are adjusted to a standard fish of trophic level 4.0 and 26 % dry mass. Shown are the antilog data of annual means of log-transformed concentrations after normalization. The *lines* represent the results of the linear regression (*solid* for significant linear trend, *dashed* for not significant) and the LOESS smoother (*solid* for significant non-linear trend, *dashed* for not significant). *The shaded areas* mark the 95 % confidence intervals of the LOESS function. Data basis: ESB: annual pool samples of approximately 20 bream each; FS: individual fish of different species. *Red line* EQS_Biota_ (20 μg/kg wet weight)

Trend analysis revealed significant decreasing trends (linear trends, *p* < 0.01) for Hg in all ESB samples and levels of complexity (i.e., samples from all ESB sites in German freshwaters (level 1), samples from all ESB sites in the Elbe (level 2), and samples from the ESB site Prossen at Elbe km 13 (level 3)). Analysis of the FS data was performed for the whole data set (including all species) and for bream (Online Resource, Tables S8, S9, S10). Decreasing Hg trends were only detected when focusing on bream but not when data of all fish species were included in the trend analysis (Fig. 2). Similarity between the relatively homogeneous ESB data and the FS data obviously increased with a reduction in sample variability.

The difference between species is also evident when comparing the relative standard deviation (in %) of the normalized data sets. With respect to the Elbe sampling site Prossen and the years 1996–2013, the relative standard deviation of calculated mean Hg concentrations in fish samples including all fish species ranged between 49 and 128 % (mean 81 %) depending on year, whereas it was only 0.5–51 % (mean 22 %) when only bream were considered.

#### Perfluorooctane sulfonic acid

PFOS was analyzed in ESB samples of the years 1995–2010 (Theobald et al. 2011) and 2013–2014. Adjustment to TL 4 using a TMF of 3.6 resulted in higher PFOS levels because bream occupy a trophic level below 4.0. Reported concentrations during the entire study period ranged between 0.3 and 91 μg/kg *w*/*w*, which correspond to normalized concentrations of 1.4–320 μg/kg *w*/*w* (Online Resource, Table S11). Lowest values were detected in Lake Belau and highest in the Rhine and at the lower Elbe site Blankenese. If compliance was based on normalized concentrations, only fish from Lake Belau met the EQS_Biota_ of 9.1 μg/kg during the entire study period.

When considering only the period 2005–2010 (the time span for which also FS data were available), PFOS in ESB bream was in the range of 0.6–70 μg/kg, respectively, 2.3–234 μg/kg when normalized to 26 % dry mass and TL 4 (Table 2).

PFOS data from the FS monitoring were available only for rivers in Bavaria (*n* = 16, Van de Graaf et al. 2008) and North Rhine-Westphalia (*n* = 1252) covering the years 2005 and 2006–2010, respectively.

In contrast to the HCB and Hg data, which originate mostly from regular surveillance monitoring programs of the federal states, PFOS was analyzed within various programs addressing different questions. This included, for instance, the operational and investigative monitoring in highly contaminated waters. Accordingly, the PFOS data vary widely.

Similar to the ESB data, adjustment to TL 4 resulted in higher PFOS levels for 77 % of the fish. Reported concentrations ranged between <0.2 and 2749 μg/kg *w*/*w*, corresponding to normalized concentrations of <0.8–4852 μg/kg *w*/*w* (Table 2, and Online Resource, Table S12). Highest reported PFOS levels above 500 μg/kg were detected in a small creek in North Rhine-Westphalia, which is highly contaminated by a known point source, and in ponds in North Rhine-Westphalia with unknown water supply. In contrast, relatively low PFOS concentrations were found in fish from the large streams Danube and Rhine (North Rhine-Westphalian section), i.e., 4.9–9.9 μg/kg (normalized: 5.6–11.4 μg/kg) in the Danube in 2005, and 2.6–72 μg/kg (normalized: 3.0–236 μg/kg) in the Rhine in 2006–2010. These values are lower or in the same range as the ESB data for Danube (2005, reported 14.5–33 μg/kg; normalized 54–106 μg/kg) and Rhine (2006–2010, reported 0.6–70 μg/kg; normalized: 2.3–235 μg/kg). Based on the normalized data, only two sampling sites of the FS monitoring programs met the EQS_Biota_ of 9.1 μg/kg *w*/*w* in 2010 (i.e., Urft /“Urfttalsperre” and Große Aue/“an der Landesgrenze”).

**Fig. 3:**
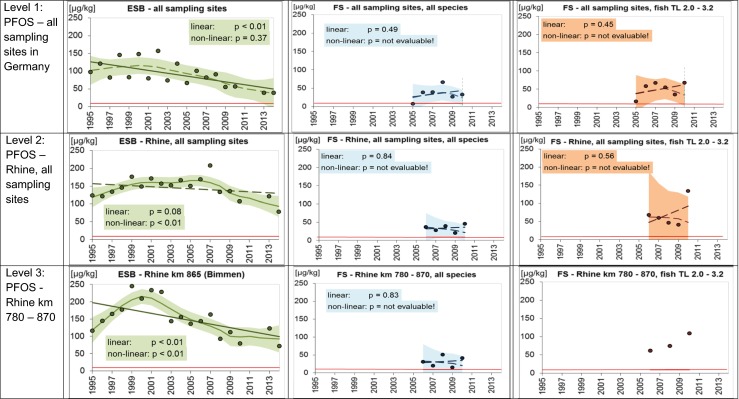
Temporal trends in PFOS concentrations (μg/kg; wet weight-based) in muscle tissue of freshwater fish in Germany sampled by the German Environmental Specimen Bank (ESB) and the federal states (FS). Data are adjusted to a standard fish of trophic level 4.0 and 26 % dry mass. Shown are the antilog data of annual means of log-transformed concentrations after normalization. The *lines* represent the results of the linear regression (*solid* for significant linear trend, *dashed* for not significant) and the LOESS smoother (*solid* for significant non-linear trend, *dashed* for not significant). The *shaded areas* mark the 95 % confidence intervals of the LOESS function. Data basis: ESB: annual pool samples of approximately 20 bream each; FS: individual fish of different species. *Red line* EQS_Biota_ (9.1 μg/kg wet weight)

Trend analysis indicates a decrease in PFOS in the ESB samples (Fig. 3). Trends, however, were not significant when considering only the period 2005–2010. When extending trend analysis to the years 1995–2014, a significant decreasing trend (linear trend, *p* < 0.01) was detected for the combined data of all ESB freshwater sites (level 1).

Regarding the FS data from 2005 to 2010, trend analysis was performed for data of all fish species and for fish of TL 2.0–3.2 (i.e., trophic levels similar to bream) because not enough bream data were available for direct species comparison (Online Resource, Tables S12, S13, S14). The PFOS data referring to fish from ponds with unknown water supply and from the small highly contaminated creek were not included in trend analysis to avoid a bias. No significant trends were detected in the final data set. In contrast to the ESB data, PFOS seems to increase in fish sampled by the FS when data of all German sites are analyzed together (level 1). However, as mentioned above, these results are influenced by the heterogeneity of the sampling programs.

In view of the available FS data, further comparison between the ESB and the FS data focused on the Rhine. The FS data, however, refer to the lower Rhine (km 640–870) only, while the ESB data also include sampling sites in the upper and middle Rhine. Trend analysis including all Rhine sampling sites (Fig. 3, level 2) indicates that mean PFOS levels in the ESB samples have decreased between 1995 and 2014, but trends were not significant. PFOS in FS samples comprising all fish species were lower and remained more or less constant between 2006 and 2010. These lower concentrations are, at least in part, related to the normalization to TL 4, because 66 % of the fish collected by the FS belong to higher trophic levels than bream, which means that normalization had less effect on the PFOS levels. Similarly, no significant trend was observed when considering only fish of TL 2.0–3.2.

When focusing trend analysis on only one sampling site (level 3), decreasing PFOS levels were observed in the ESB samples from Bimmen (Rhine km 865) (Fig. 3, level 3). The trend, however, was only significant when all sampling years (1995–2014) were included in trend analysis (linear trend, *p* < 0.01) while no significance was detected for the period 2006–2010. No temporal trend was observed for fish sampled by the FS in the Rhine section km 780–860 (Fig. 3, all species). The picture here is quite similar to level 2 because the data overlap to more than 50 %. No trend analysis was possible for fish of TL 2.0–3.2 sampled between Rhine km 780 and 870 because the data cover only 3 years (Online Resource, Table S14).

Intra-annual variability of PFOS levels in fish of different species sampled between Rhine km 780 and 870 was relatively high (Online Resource, Table S14). The standard deviations of the normalized data ranged between 31 and 102 % (mean 73 %) depending on year. When considering only fish of TL 2.0–3.2, relative standard deviations ranged between 36 and 86 % (mean 68 %) and were thus only slightly lower. Since the database for PFOS is relatively small, it can only be speculated about the reasons for the observed variability. One possible reason is that normalization—at least when based on default values as done here—cannot overcome species-specific differences in PFOS uptake, excretion, metabolism, and/or accumulation. Another reason may be that PFOS levels differed between the sampling sites located between Rhine km 780 and km 870, which were evaluated together in order to obtain enough data for trend analysis.

### Discussion of normalization procedure

The present study integrates fish monitoring data of different monitoring programs in Germany. We compared relatively homogeneous ESB data referring to annual pooled samples of bream muscle with muscle samples of individual fish of different species analyzed by the federal states. In order to compare these data in a meaningful way, it was necessary to standardize the reported concentrations to reduce biases caused, e.g., by different accumulation behavior between fish of different species and size, and overcome the effect of biomagnification in the food web (EC 2014).

The new EU Guidance Document No. 32 (EC 2014) recommends normalization to a predatory fish belonging to trophic level 4.0 with 5 % lipid and 26 % dry mass. Trophic level, lipid content, and dry mass are not only species-specific but vary between individuals and ecosystems. The EC guidance document, therefore, recommends basing the normalization on measured site- and fish-specific data. If measured values are not available, the guidance document suggests to use default values, for instance from FishBase (Froese and Pauly 2016).

This pragmatic approach is applicable to all priority substances, in contrast to, e.g., the procedure used by Åkerblom et al. (2014) who converted measured Hg concentrations in fish from Swedish lakes to a standard pike of 1 kg fresh weight. This Hg-specific approach based on Meili et al. (2004) relies on an empirically supported transfer function and default values derived from a database for Nordic fish (Munthe et al. 2004). Similar to the EU procedure, the measured Hg data are adjusted to a common trophic level.

In the present study, normalization followed the recommendations of the Guidance Document No. 32 (EC 2014). Since no measured values were available for trophic levels and, in the case of FS data, for dry mass, normalization had to resort to default values from FishBase (EC 2014; Froese and Pauly 2016).

The results for Hg show that similarity between the relatively homogeneous ESB data and the FS data increases when sample diversity is reduced (i.e., FS samples including all fish species compared to FS samples of bream only). These findings indicate that the adjustment to a common trophic level was not successful. A likely reason for this is the use of default TL values, which do not adequately reflect the actual trophic level of the respective fish.

For HCB and PFOS, the databases were relatively small and allowed no sound conclusion regarding the usefulness of normalization for data reporting under the WFD.

In order to evaluate the effectiveness of the normalization procedure more closely, we analyzed the data of selected sampling sites where fish of different species had been sampled in the same year. It was assumed that the basic exposure at the sites was similar for all species and normalization of the fish data would thus reduce the variability, leading to a decline in relative standard deviation. Table 3 summarizes the data.

**Table 3:** Comparison of the relative standard deviations (%SD) before and after normalization. Data refer to annual mean HCB, Hg, and PFOS concentrations determined in muscle tissue of individual fish of different species at selected sampling sites. Normalization step 1: HCB: normalized to 5 % lipid based on measured lipid concentrations; Hg + PFOS: normalized to 26 % dry mass (DM) based on default DM values (FishBase); normalization step 2: lipid, respectively, DM normalization + adjustment to trophic level (TL) 4.0 based on default TL values (FishBase)

Substance	Sampling site	Year	N_fish_	N_species_	Relative standard deviation (%)		
					reported conc.	Step 1 (26 % DM/5 % lipid)	Step 2 (DM/lipid + TL)
HCB	Danube/Bad Abbach	2000	6	5	208	127	118
	Eger/Egermühle	2004	7	2	88	15	38
	Lippe/Lippholthausen	2004	12	3	112	65	77
Hg	Elbe/Meißen	1999	33	3	62	63	48
	Elbe/Gallin	1999	40	3	42	43	45
	Elbe/Wahrenberg	1999	41	3	57	56	37
	Elbe/Prossen	2013	20	7	67	66	49
	Elbe/Dresden-Pieschen	2013	20	5	51	49	48
	Elbe/Meißen	2013	20	7	67	67	76
	Elbe/Strehla	2013	20	7	91	89	76
	Elbe/Belgern	2013	20	4	56	54	32
	Freiberger Mulde/uh Podelwitz	2013	8	3	54	55	82
PFOS	Möhne/Möhnetalsperre	2006	36	7	116	117	112
	Ruhr/Hattingen	2006	12	5	93	94	73
	Rhein/Rees	2006	14	7	44	45	81
	Rhein/uh Ruhrmündung	2007	6	3	39	42	31
	Rhein/oh Ruhrmündung	2007	8	4	72	73	45
	Möhne/Möhnetalsperre	2008	12	4	41	41	70
	Lenne/Pegel Hohenlimburg	2008	6	4	58	58	42
	Möhne/Möhnetalsperre	2010	16	4	73	71	50

The results are very heterogeneous and demonstrate that the normalization is not generally effective in reducing the variability between species.

Normalization of the HCB data used measured lipid levels and default values for TLs. The approach was limited to three sampling sites where enough fish of different species had been sampled in the same year and where HCB levels were above the LOQ. Normalization to 5 % lipid content and TL 4 reduced data heterogeneity in all cases by 31–57 %. However, in two cases, lipid normalization alone had stronger effects than combined lipid and TL normalization.

Hg and PFOS data were normalized using default values for dry mass and TL. For both substances, normalization to 26 % dry mass alone had very little effects. Combined normalization of the Hg data to 26 % dry mass and TL 4 resulted in reduced data variability by 6–43 % in six of nine cases while data heterogeneity increases in three cases. Results for PFOS are quite similar with reduced data variability by 3–38 % in six of eight cases while combined normalization strongly enhanced variability in two cases.

These results indicate that the normalization procedure proposed in the WFD Guidance Document No. 32 on biota monitoring (EC 2014) may be feasible for rather simple lipophilic compounds like HCB. However, it might oversimplify the real situation for substances like Hg and PFOS that behave in a more complicated manner (i.e., binding to sulfhydryl groups of proteins or to proteins in general).

Moreover, the findings question the relevance of normalizing chemical monitoring data based on default values. The fact that strong positive effects (i.e., reduction of variability) were obtained when normalization was based on measured values (i.e., lipid content in the case of HCB) underlines the importance of including the measurement of dry mass and lipid content in fish monitoring programs. Similarly, site-specific trophic levels of fish (determined, e.g., from stable isotope ratios against reference organisms like mussels) are required for the adjustment to a common trophic level. Furthermore, the derivation of TMFs should be standardized, and more TMF data from riverine systems are needed. This is especially important in the case of Hg where a strong dependency of trophic magnification on physical and chemical parameters like pH, DOC, and productivity is reported (Lavoie et al. 2013; Clayden et al. 2013). The generic application of the same TMF for different waters may therefore lead to erroneous results.

Taken together, a reconsideration of the recommended normalization approach may be necessary which refrains from using default values. Furthermore, preconditions for normalization should be defined. Lipid normalization, for instance, should only be applied on substances for which a relationship between lipid content and contaminant level is given (Hebert and Keenleyside 1995).

## Conclusions

The present study gives an overview of the existing fish monitoring data for HCB, Hg, and PFOS in German freshwaters generated under different programs.

Hg and HCB are monitored for many years now, and the existing data provide a coherent picture of the contamination of freshwater fish. According to these data, levels of Hg are still high in German freshwaters and exceeded the EQS_Biota_ by far at all sampling sites in 2013. However, concentrations in fish are decreasing at most sites. HCB levels in fish have also decreased in the last decade and are in the range of the EQS_Biota_ or even below at most FS sampling sites while exceedance is still detected at most ESB sites.

Considerably fewer data are available for PFOS so that a general statement on the pollution situation in German freshwaters is not easy. According to the ESB data, concentrations in fish have decreased in the last years, but the EQS_Biota_ for PFOS is still exceeded in the main German streams Elbe, Danube, and Rhine as well as in their tributaries Mulde, Saale, and Saar. The FS data come mostly from North Rhine-Westphalia and were generated in programs addressing different questions. Accordingly, the heterogeneity of the data is high, and general conclusions cannot be drawn.

Furthermore, we wanted to evaluate in how far data treatment can replace a standardized sampling strategy. For this purpose, we compared the data of the ESB (representing a highly standardized sampling strategy) with the heterogeneous FS data after data treatment according to the recommendations of the WFD Guidance Document No. 32. However, normalization had to rely mainly on default values because measured values for dry mass were not available for the FS data and neither the ESB nor the FS had determined the trophic level of the sampled fish.

Based on our results for Hg, it can be concluded that the data treatment proposed in the Guidance Document No. 32 may be a helpful tool when managing existent data and linking these data to newly generated ones. The normalization, however, should rely exclusively on measured data for dry mass, lipid content, and trophic level.

In our study, the adjustment using default values for dry mass and trophic levels could not overcome individual and species-specific differences in accumulation.

We therefore conclude that a commitment to a harmonized sampling strategy is inevitable to ensure that compliance monitoring results in a comparable assessment at different sites. Since it will not be possible to sample the same fish species at all locations, there should be an agreement on a pick list of only a few species (as, e.g., proposed by RAKON 2015).

In trend monitoring, a highly standardized sampling strategy is considered to be essential. A monitoring strategy may include different fish species at different sampling locations. At a specific location, however, fish of the same species and size (respectively, age) should be collected during the same season at every sampling interval. It may be desirable to sample routinely more than one species per site because accumulation behavior and body burdens differ between species (EC 2014). Since this may lead to different trends at the same location, it is important to agree upon how these data are aggregated and used for trend assessment.

Finally, effort should be made to improve the normalization procedures. Only reliable data normalization will allow a sound comparison of data from different regional and national monitoring programs. If this is accomplished, then data from highly standardized programs, as, e.g., the ESB, can help to interpret data from other biomonitoring programs, and data of different species can be integrated into an overall assessment.

## Electronic supplementary material

ESM 1 (DOCX 103 kb)
