# Supplementary material for: Biota monitoring and the Water Framework Directive—can normalization overcome shortcomings in sampling strategies?
Source: Environ Sci Pollut Res Int. 2016 Aug 18;23(21):21927–39. doi: 10.1007/s11356-016-7442-2 (PMC5099357; doi:10.1007/s11356-016-7442-2)
Supplement: Supplementary file 1 — (DOCX 103 kb) [file 11356_2016_7442_MOESM1_ESM.docx]

**Biota monitoring and the Water Framework Directive – can normalization overcome shortcomings in sampling strategies?**

Annette Fliedner*, Heinz Rüdel, Diana Teubner, Georgia Buchmeier, Jaqueline Lowis, Christiane Heiss, Jörg Wellmitz, Jan Koschorreck

* [annette.fliedner@ime.fraunhofer.de](mailto:annette.fliedner@ime.fraunhofer.de); Fraunhofer Institute for Molecular Biology and Applied Ecology (Fraunhofer IME), 57392 Schmallenberg, Germany.

**Supplementary Material**

Table S1: Trophic levels (based on diet studies) and dry mass values from FishBase (Froese and Pauly 2016) of the most commonly monitored fish species

| **Species** | | **Trophic level** | **Dry mass*** |
| --- | --- | --- | --- |
| **Latin name** | **English name** |  |  |
| *Abramis brama* | bream | 3.1 | 0.248 |
| *Anguilla anguilla* | eel | 3.6 | 0.378 |
| *Barbus barbus* | barb | 3.1 | *0.25* |
| *Blicca bjoerkna* | white bream | 3.2 | 0.266 |
| *Carassius carassius* | Crucian carp | 3.1 | *0.25* |
| *Carassius gibelio* | Prussian carp | 2.5 | *0.25* |
| *Chondrostoma nasus* | common nase | 2.0 | *0.25* |
| *Coregonus spec.* | whitefish | 3.1 | *0.25* |
| *Cyprinus carpio* | carp | 3.1 | 0.194 |
| *Esox lucius* | pike | 4.1 | 0.238 |
| *Hucho hucho* | huchen | 4.2 | *0.25* |
| *Leuciscus aspius* | asp | 4.5 | 0.259 |
| *Leuciscus idus* | orf | 3.8 | *0.25* |
| *Leuciscus leuciscus* | dace | 2.9 | *0.25* |
| *Lota lota* | burbot | 3.8 | *0.25* |
| *Oncorhynchus mykiss* | rainbow trout | 4.1 | 0.314 |
| *Perca fluviatilis* | perch | 4.4 | 0.246 |
| *Rutilus rutilus* | roach | 3.0 | 0.249 |
| *Salmo trutta* | trout | 3.4 | 0.235 |
| *Salvelinus alpinus* | char | 3.3 | *0.25* |
| *Sander lucioperca* | pikeperch | 4.0 | 0.255 |
| *Scardinius erythrophthalmus* | rudd | 2.9 | 0.255 |
| *Silurus glanis* | catfish | 4.4 | *0.25* |
| *Squalius cephalus* | chub | 2.7 | *0.25* |
| *Thymallus thymallus* | grayling | 3.1 | *0.25* |
| *Tinca tinca* | tench | 3.7 | 0.191 |

* If more than one value was provided for dry mass, the value referring to ‘lakes’ was used. In the case of missing dry mass data a value of 0.25 was applied (in italics).

Table S2: δ^15^N values in bream (*Abramis brama*) at sampling sites of the German Environmental Specimen Bank and calculated trophic levels (TL) using δ^15^N data for suspended particulate matter (SPM) as baseline (assumed TL = 1.0). Data refer to annual pooled muscle samples.

| **Sampling site** | **year** | **δ^15^N_fish_ (‰)** | **δ^15^N_SPM_ (‰)** | **Calculated TL values** | **Mean TL** |
| --- | --- | --- | --- | --- | --- |
| Elbe / Prossen | 2006 | 13.45 | 6.36 | 3.1 | 3.1 |
|  | 2007 | 13.81 | 7.20 | 2.9 |  |
|  | 2008 | 13.84 | 6.45 | 3.2 |  |
|  | 2009 | 14.08 | 6.30 | 3.3 |  |
| Elbe / Cumlosen | 2006 | 13.61 | 7.02 | 2.9 | 2.9 |
|  | 2007 | 14.33 | 7.88 | 2.9 |  |
|  | 2008 | 13.86 | 7.81 | 2.8 |  |
|  | 2009 | 13.86 | 7.34 | 2.9 |  |
| Elbe /Blankenese | 2006 | 14.38 | 8.22 | 2.8 | 3.0 |
|  | 2007 | 15.09 | 7.95 | 3.1 |  |
|  | 2008 | 15.27 | 8.13 | 3.1 |  |
|  | 2009 | 14.96 | 8.21 | 3.0 |  |
| Mulde / Dessau | 2006 | 15.10 | 8.76 | 2.9 | 2.8 |
|  | 2007 | 15.94 | 9.31 | 2.9 |  |
|  | 2008 | 15.61 | 9.51 | 2.8 |  |
|  | 2009 | 15.39 | 9.82 | 2.6 |  |
| Saale / Wettin | 2006 | 14.54 | 6.95 | 3.2 | 3.5 |
|  | 2007 | 15.28 | 6.62 | 3.5 |  |
|  | 2008 | 15.68 | 6.56 | 3.7 |  |
|  | 2009 | 15.20 | 6.97 | 3.4 |  |

Table S3: **Environmental Specimen Bank: HCB** concentrations (µg/kg wet weight) in bream (*Abramis brama*). Data refer to annual pooled samples of approx. 20 fish each. Left side: HCB concentrations normalized to 5% lipid content and adjusted to trophic level 4.0 (TL 4). Right side: original reported concentrations. EQS: 10 µg/kg ww.

| **Water-body** | **sampling site** | **sampling period** | **n** | **HCB normalized to standard fish of 5% lipid and TL 4** | | | | | | | **reported HCB concentrations** | | | | |
| --- | --- | --- | --- | --- | --- | --- | --- | --- | --- | --- | --- | --- | --- | --- | --- |
|  |  |  |  | mean | ± SD | median | min | max | Linear trend | EQS met in 2013 | mean | ± SD | median | min | max |
| Lake Belau | Lake Belau | 1997-2013 | 9 | 2.3 | 1.4 | 1.7 | 1.3 | 5.7 | no | yes | 0.2 | 0.1 | 0.2 | < 0.2 | 0.4 |
| Saar | Güdingen | 1994-2013 | 20 | 10.1 | 3.4 | 9.9 | 4.7 | 17.7 | decrease: -0.4 µg/kg p.a.; p<0.01 | yes | 3.6 | 1.0 | 3.5 | 1.7 | 5.7 |
|  | Rehlingen | 1994-2013 | 20 | 9.4 | 2.9 | 8.7 | 5.1 | 14.8 | decrease: -0-2 µg/kg p.a.; p=0.03 | yes | 3.3 | 1.1 | 3.3 | 1.4 | 5.2 |
| Rhine | Weil | 1995-2009; 2011-2012 | 17 | 19.8 | 10.2 | 15.4 | 8.2 | 44.7 | decrease: -1.7 µg/kg p.a.; p<0.01 | no* | 9.4 | 4.2 | 8.3 | 3.5 | 15.4 |
|  | Iffezheim | 1995-2013 | 19 | 62.9 | 27.7 | 57.4 | 32.7 | 128.8 | decrease: -3.1 µg/kg p.a.; p<0.01 | no | 26.0 | 11.0 | 25.0 | 13.8 | 50.1 |
|  | Koblenz | 1995-2013 | 19 | 40.5 | 22.8 | 32.7 | 19.8 | 101.0 | decrease: -3.0 µg/kg p.a.; p<0.01 | no | 10.7 | 3.4 | 10.2 | 4.9 | 18.1 |
|  | Bimmen | 1995-2013 | 19 | 31.9 | 14.1 | 29.2 | 13.8 | 66.4 | decrease: -2.0 µg/kg p.a.; p<0.01 | no | 8.4 | 3.3 | 8.1 | 3.3 | 14.8 |
| Elbe | Prossen | 1993-2013 | 21 | 231.2 | 176.6 | 185.3 | 37.9 | 609.9 | decrease: -26.4 µg/kg p.a.; p<0.01 | no | 37.0 | 26.5 | 34.0 | 10.1 | 91.1 |
|  | Zehren | 1993-2013 | 21 | 410.7 | 328.6 | 322.3 | 68.9 | 1183 | decrease: -48.9 µg/kg p.a.; p<0.01 | no | 51.8 | 37.5 | 42.7 | 12.2 | 151.8 |
|  | Barby | 1993-2013 | 21 | 250.4 | 193.7 | 185.2 | 44.6 | 740.1 | decrease: -25.4 µg/kg p.a.; p<0.01 | no | 47.1 | 42.3 | 29.0 | 8.0 | 157.6 |
|  | Cumlosen | 1993-2013 | 21 | 189.4 | 147.9 | 128.1 | 42.6 | 523.7 | decrease: -19.8 µg/kg p.a.; p<0.01 | no | 34.9 | 22.8 | 21.0 | 13.5 | 88.4 |
|  | Blankenese | 1993-2013 | 21 | 104.2 | 86.3 | 69.2 | 26.0 | 328.2 | decrease: -11.4 µg/kg p.a.; p<0.01 | no | 32.8 | 22.0 | 26.5 | 10.6 | 85.7 |
| Saale | Wettin | 1995-2013 | 19 | 21.2 | 17.4 | 17.8 | 9.5 | 88.3 | decrease: -1.7 µg/kg p.a.; p<0.01 | yes | 6.6 | 2.4 | 6.0 | 2.7 | 12.4 |
| Mulde | Dessau | 1995-2013 | 19 | 216.2 | 217.5 | 111.5 | 58.2 | 788.7 | decrease: -30.1 µg/kg p.a.; p<0.01 | no | 30.9 | 27.0 | 18.0 | 11.1 | 110.0 |
| Danube | Ulm | 2004-2013 | 10 | 3.5 | 0.6 | 3.5 | 2.5 | 4.5 | decrease: -0.1 µg/kg p.a.; p=0.04 | yes | 1.4 | 0.3 | 1.2 | 1.1 | 2.0 |
|  | Kelheim | 2004-2013 | 10 | 9.0 | 2.7 | 8.8 | 3.7 | 12.8 | no | yes | 3.1 | 1.1 | 3.5 | 0.7 | 4.3 |
|  | Jochenstein | 2004-2013 | 10 | 6.1 | 1.3 | 6.2 | 3.5 | 7.8 | no | yes | 2.6 | 0.9 | 2.5 | 1.5 | 4.1 |

*refers to 2012

Table S4: **Federal States Monitoring: HCB** concentrations (µg/kg wet weight) in fish from **German freshwaters (all sampling sites).** Data refer to individual fish. Left side: Concentrations normalized to trophic level 4.0 (TL 4) and 5% lipid content. Right side: original reported concentrations.

| **year** | **n** | **HCB concentrations normalized to 5% lipid and TL 4** | | | | | **reported HCB concentrations** | | | | |
| --- | --- | --- | --- | --- | --- | --- | --- | --- | --- | --- | --- |
|  |  | mean | ± SD | median | min | max | mean | ± SD | median | min | max |
| **all species** | | | | | | | | | | | |
| 2000 | 90 | 29.6 | 62.2 | 11.9 | 0.4 | 361 | 25.7 | 142 | 2.0 | < 0.2 | 1331 |
| 2001 | 37 | 16.8 | 34.7 | 5.1 | 1.1 | 200 | 43.7 | 82.4 | 16.8 | 3.6 | 470 |
| 2002 | 294 | 18.9 | 23.0 | 16.7 | < 0.2 | 262 | 51.4 | 32.5 | 50.0 | < 0.2 | 282 |
| 2003 | 211 | 9.9 | 22.8 | 4.3 | < 0.2 | 262 | 18.2 | 75.8 | 2.1 | < 0.2 | 984 |
| 2004 | 222 | 7.9 | 17.9 | 3.5 | < 0.2 | 168 | 5.4 | 10.2 | 0.9 | < 0.2 | 93.5 |
| 2005 | 137 | 14.7 | 53.2 | 7.3 | 0.43 | 591 | 22.0 | 21.5 | 12.8 | < 0.2 | 116 |
| 2006 | 75 | 10.8 | 35.3 | 3.0 | < 0.2 | 264 | 12.3 | 19.7 | 7.3 | < 0.2 | 104 |
| 2007 | 55 | 6.3 | 13.1 | 3.3 | 0.7 | 84.7 | 11.8 | 12.2 | 8.2 | 1.8 | 69.5 |
| 2008 | 98 | 5.4 | 9.05 | 2.53 | 0.44 | 55.6 | 7.3 | 17.5 | 4.2 | < 0.2 | 132 |
| 2009 | 77 | 9.9 | 9.7 | 7.8 | 0.21 | 59.2 | 25.1 | 21.4 | 19.7 | 0.8 | 151 |
| 2010 | 97 | 8.0 | 5.7 | 6.4 | 0.83 | 27.1 | 8.3 | 17.2 | 2.5 | 1.1 | 93.9 |
| **eel** | | | | | | | | | | | |
| 2000 | 20 | 31.0 | 72.7 | 4.64 | 1.13 | 328 | 102 | 293 | 14.3 | 1.94 | 1331 |
| 2001 | 37 | 16.8 | 34.7 | 5.06 | 1.12 | 200 | 43.6 | 82.4 | 16.8 | 3.60 | 470 |
| 2002 | 283 | 16.0 | 8.99 | 16.6 | < 0.2 | 98.0 | 53.0 | 31.9 | 51.5 | < 0.2 | 282 |
| 2003 | 104 | 12.4 | 29.7 | 4.69 | 0.35 | 262 | 36.1 | 105 | 9.64 | 0.50 | 984 |
| 2004 | 90 | 5.45 | 7.58 | 3.22 | 0.61 | 50.4 | 12.0 | 13.2 | 7.95 | 2.40 | 93.5 |
| 2005 | 113 | 8.57 | 6.10 | 8.20 | 0.43 | 27.8 | 26.1 | 21.3 | 16.3 | 0.31 | 116 |
| 2006 | 68 | 8.94 | 32.3 | 2.99 | 0.18 | 264 | 13.0 | 20.2 | 7.73 | < 0.2 | 104 |
| 2007 | 55 | 6.33 | 13.1 | 3.19 | 0.70 | 84.7 | 11.8 | 12.2 | 8.20 | 1.78 | 69.5 |
| 2008 | 65 | 5.04 | 9.19 | 2.47 | 0.63 | 55.6 | 10.7 | 20.8 | 5.55 | 0.50 | 132 |
| 2009 | 77 | 9.91 | 9.71 | 7.82 | 0.21 | 59.2 | 25.1 | 21.4 | 19.7 | 0.80 | 151 |

Table S5: **Federal States Monitoring: HCB** concentrations (µg/kg wet weight) in fish from the **river Danube (all sampling sites)**. Data refer to individual fish. Left side: Concentrations normalized to trophic level 4.0 (TL 4) and 5% lipid content. Right side: original reported concentrations.

| **year** | **n** | **HCB concentrations normalized to 5% lipid and TL 4** | | | | | **reported HCB concentrations** | | | | |
| --- | --- | --- | --- | --- | --- | --- | --- | --- | --- | --- | --- |
|  |  | mean | ± SD | median | min | max | mean | ± SD | median | min | max |
| **all species** | | | | | | | | | | | |
| 2000 | 15 | 90.0 | 134 | 36.6 | 5.12 | 361 | 118 | 340 | 4.86 | 1.13 | 1331 |
| 2001 | 6 | 56.7 | 73.6 | 29.4 | 4.50 | 200 | 131 | 171 | 73.4 | 14.3 | 470 |
| 2002 | 2 | 5.69 | 3.31 | 5.69 | 3.34 | 8.03 | 22.7 | 7.64 | 22.7 | 17.3 | 28.1 |
| 2003 | 20 | 18.8 | 57.5 | 4.65 | 0.58 | 262 | 56.5 | 219 | 1.31 | < 0.2 | 984 |
| 2004 | 17 | 9.76 | 13.4 | 5.04 | 2.45 | 54.1 | 11.1 | 22.3 | 1.04 | 0.28 | 93.5 |
| 2005 | 14 | 8.73 | 9.87 | 5.46 | 1.30 | 34.3 | 16.1 | 29.7 | 9.24 | < 0.2 | 116 |
| 2006 | 12 | 7.99 | 12.8 | 3.55 | 1.22 | 46.8 | 22.2 | 30.2 | 10.9 | 3.75 | 104 |
| 2007 | 7 | 3.44 | 1.21 | 3.19 | 1.76 | 5.10 | 9.72 | 3.31 | 10.7 | 5.00 | 14.2 |
| 2008 | 15 | 5.97 | 8.94 | 2.92 | 1.90 | 37.6 | 12.3 | 33.3 | 4.72 | < 0.2 | 132 |
| 2009 | 5 | 5.68 | 5.36 | 4.21 | 1.78 | 15.0 | 14.2 | 15.1 | 6.15 | 5.20 | 40.6 |
| **eel** | | | | | | | | | | | |
| 2000 | 3 | 149 | 156 | 72.3 | 45.8 | 328 | 543 | 683 | 189 | 110 | 1331 |
| 2001 | 6 | 56.7 | 73.6 | 29.4 | 4.50 | 200 | 131 | 171 | 73.4 | 14.3 | 470 |
| 2002 | 2 | 5.69 | 3.31 | 5.69 | 3.34 | 8.03 | 22.7 | 7.64 | 22.7 | 17.3 | 28.1 |
| 2003 | 8 | 39.1 | 90.3 | 5.90 | 3.28 | 262 | 140 | 341 | 13.4 | 4.96 | 984 |
| 2004 | 6 | 8.22 | 10.1 | 4.56 | 2.48 | 28.7 | 25.2 | 33.6 | 12.9 | 6.50 | 93.5 |
| 2005 | 9 | 7.28 | 8.00 | 5.18 | 2.04 | 27.8 | 24.4 | 34.9 | 12.2 | 8.00 | 116 |
| 2006 | 12 | 7.99 | 12.8 | 3.55 | 1.22 | 46.8 | 22.2 | 30.2 | 10.9 | 3.75 | 104 |
| 2007 | 7 | 3.44 | 1.21 | 3.19 | 1.76 | 5.10 | 9.72 | 3.31 | 10.7 | 5.00 | 14.2 |
| 2008 | 8 | 6.83 | 12.4 | 2.38 | 1.90 | 37.6 | 22.7 | 44.2 | 7.26 | 4.72 | 132 |
| 2009 | 5 | 5.68 | 5.36 | 4.21 | 1.78 | 15.0 | 14.2 | 15.1 | 6.15 | 5.20 | 40.6 |

Table S6: **Federal States Monitoring: HCB** concentrations (µg/kg wet weight) in fish from the **Danube sampling sites km 2434 - 2400**. Data refer to individual fish. Left side: Concentrations normalized to trophic level 4.0 (TL 4) and 5% lipid content. Right side: original reported concentrations.

| **year** | **n** | **HCB concentrations normalized to 5% lipid and TL 4** | | | | | **reported HCB concentrations** | | | | |
| --- | --- | --- | --- | --- | --- | --- | --- | --- | --- | --- | --- |
|  |  | mean | ± SD | median | min | max | mean | ± SD | median | min | max |
| **all species** | | | | | | | | | | | |
| 2000 | 8 | 105 | 149 | 40.0 | 5.12 | 361 | 192 | 462 | 8.905 | 1.80 | 1331 |
| 2001 | 4 | 75.8 | 86.3 | 47.1 | 9.31 | 200 | 161 | 207 | 73.4 | 27.6 | 470 |
| 2002 | 1 | 8.03 |  | 8.03 | 8.03 | 8.03 | 28.1 |  | 28.1 | 28.1 | 28.1 |
| 2003 | 4 | 69.9 | 128 | 8.24 | 0.58 | 262 | 256 | 486 | 19.075 | 0.42 | 984 |
| 2004 | 6 | 20.0 | 19.4 | 13.9 | 3.53 | 54.1 | 22.8 | 35.5 | 10.6 | 0.35 | 93.5 |
| 2005 | 4 | 19.7 | 13.5 | 18.5 | 7.29 | 34.3 | 36.4 | 54.7 | 14.6 | 0.23 | 116 |
| 2006 | 3 | 22.6 | 21.6 | 15.3 | 5.54 | 46.8 | 60.8 | 44.6 | 63.6 | 14.9 | 104 |
| 2007 | 2 | 2.48 | 1.01 | 2.48 | 1.76 | 3.19 | 6.99 | 2.81 | 6.99 | 5.00 | 8.97 |
| 2008 | 6 | 10.1 | 13.7 | 4.77 | 2.47 | 37.6 | 23.8 | 53.1 | 0.43 | < 0.2 | 132 |
| 2009 | 3 | 7.00 | 7.04 | 4.21 | 1.78 | 15.0 | 17.5 | 20.0 | 6.15 | 5.88 | 40.6 |
| **eel** | | | | | | | | | | | |
| 2000 | 2 | 187 | 199 | 187 | 45.8 | 328 | 721 | 863 | 721 | 110 | 1331 |
| 2001 | 4 | 75.8 | 86.3 | 47.1 | 9.31 | 200 | 161 | 207 | 73.4 | 27.6 | 470 |
| 2002 | 1 | 8.03 |  | 8.03 | 8.03 | 8.03 | 28.1 |  | 28.1 | 28.1 | 28.1 |
| 2003 | 2 | 138 | 176 | 138 | 13.2 | 262 | 511 | 669 | 511 | 37.5 | 984 |
| 2004 | 2 | 16.1 | 17.8 | 16.1 | 3.53 | 28.7 | 51.7 | 59.1 | 51.7 | 9.92 | 93.5 |
| 2005 | 2 | 18.5 | 13.1 | 18.5 | 9.25 | 27.8 | 72.2 | 61.9 | 72.2 | 28.4 | 116 |
| 2006 | 3 | 22.6 | 21.6 | 15.3 | 5.54 | 46.8 | 60.8 | 44.6 | 63.6 | 14.9 | 104 |
| 2007 | 2 | 2.48 | 1.01 | 2.48 | 1.76 | 3.19 | 6.99 | 2.81 | 6.99 | 5.00 | 8.97 |
| 2008 | 2 | 20.0 | 24.8 | 20.0 | 2.47 | 37.6 | 70.9 | 86.4 | 70.9 | 9.82 | 132 |
| 2009 | 3 | 7.00 | 7.04 | 4.21 | 1.78 | 15.0 | 23.2 | 24.5 | 23.2 | 5.88 | 40.6 |

Table S7: **Environmental Specimen Bank:** **Hg** concentrations (µg/kg wet weight) in bream (*Abramis brama*). Data refer to annual pooled samples of approx. 20 fish each. Left side: Hg concentrations normalized to 26% dry mass and adjusted to trophic level 4.0 (TL 4). Right side: original reported concentrations.

| **water** | **Sampling site** | **Sampling period** | **n** | **Hg concentrations normalized to standard fish of 26% dry mass and TL 4** | | | | | | **reported Hg concentration** | | | | |
| --- | --- | --- | --- | --- | --- | --- | --- | --- | --- | --- | --- | --- | --- | --- |
|  |  |  |  | mean | ± SD | median | min | max | Linear trend | mean | ± SD | median | min | max |
| Lake Belau | Lake Belau | 1997-2013 | 9 | 146.9 | 39.3 | 140.5 | 102.4 | 218.2 | decrease: -5.6 µg/kg p.a.; p=0.01 | 29.4 | 6.5 | 28.8 | 21.1 | 38.4 |
| Saar | Güdingen | 1994-2013 | 20 | 420.2 | 55.4 | 424.3 | 304.9 | 530.1 | no | 97.3 | 12.7 | 96.5 | 72.6 | 126.2 |
|  | Rehlingen | 1994-2013 | 20 | 501.3 | 62.6 | 500.5 | 384.1 | 611.6 | no | 116.0 | 17.4 | 116.3 | 79.5 | 149.4 |
| Rhine | Weil | 1995-2013 | 17 | 670.5 | 152.9 | 694.1 | 351.9 | 885.4 | decrease: -14 µg/kg p.a.; p=0.03 | 170.3 | 42.4 | 175.0 | 85.6 | 229.2 |
|  | Iffezheim | 1995-2013 | 19 | 1001 | 157.2 | 988.8 | 665.1 | 1381 | no | 228.5 | 33.5 | 212.6 | 153.6 | 285.8 |
|  | Koblenz | 1995-2013 | 19 | 510.1 | 142.8 | 476.4 | 354.1 | 848.8 | no | 115.5 | 30.8 | 114.1 | 69.6 | 187.0 |
|  | Bimmen | 1995-2013 | 19 | 944.6 | 331.7 | 864.8 | 541.7 | 1952 | decrease: -35.7 µg/kg p.a.; p<0.01 | 199.6 | 58.4 | 189.6 | 122.6 | 363.6 |
| Elbe | Prossen | 1993-2014 | 21 | 1541 | 553.0 | 1531 | 796.4 | 2762 | decrease: -78.7 µg/kg p.a.; p<0.01 | 315.1 | 104.3 | 305.4 | 164.3 | 514.6 |
|  | Zehren | 1993-2013 | 21 | 1927 | 578.2 | 1907 | 925.7 | 3262 | decrease: -81.8 µg/kg p.a.; p<0.01 | 392.3 | 113.1 | 380.9 | 198.5 | 641.4 |
|  | Barby | 1993-2013 | 21 | 1804 | 826.2 | 1488 | 1015 | 4054 | decrease: -102 µg/kg p.a.; p<0.01 | 378.6 | 182.2 | 302.8 | 197.6 | 881.1 |
|  | Cumlosen | 1993-2013 | 21 | 1700 | 659.5 | 1442 | 1005 | 3175 | decrease: -59.7 µg/kg p.a.; p<0.01 | 353.4 | 134.7 | 307.1 | 218.0 | 635.2 |
|  | Blankenese | 1993-2013 | 21 | 802.6 | 458.6 | 552.7 | 299.1 | 1594 | decrease: -67.7 µg/kg p.a.; p<0.01 | 184.9 | 102.8 | 127.8 | 67.2 | 358.2 |
| Saale | Wettin | 1995-2013 | 19 | 1443 | 279.3 | 1499 | 910.9 | 1896 | increase: +25.8 µg/kg p.a.; p=0.01 | 338.4 | 73.8 | 368.2 | 193.7 | 447.7 |
| Mulde | Dessau | 1995-2013 | 19 | 1667 | 918.2 | 1317 | 885.3 | 3987 | decrease: -130 µg/kg p.a.; p<0.01 | 345.7 | 184.6 | 264.3 | 186.0 | 825.3 |
| Danube | Ulm | 2004-2013 | 10 | 450.3 | 69.3 | 461.6 | 311.3 | 537.4 | no | 105.4 | 19.3 | 103.5 | 73.2 | 133.7 |
|  | Kelheim | 2004-2013 | 10 | 811.7 | 267.0 | 746.1 | 477.8 | 1251 | no | 180.1 | 55.4 | 164.7 | 113.6 | 271.5 |
|  | Jochenstein | 2004-2013 | 10 | 1263 | 190.4 | 1280 | 903.9 | 1478 | no | 311.7 | 41.7 | 314.8 | 248.5 | 387.3 |

N: number of annual pool samples

Table S8: Federal states monitoring: Hg concentrations (µg/kg wet weight) in fish from German freshwaters (all sampling sites). Data refer to individual fish. Left side: Hg concentrations normalized to 26% dry mass and adjusted to trophic level 4.0 (TL 4). Right side: original reported concentrations.

| **year** | **n** | **Hg concentrations normalized to 26% DM and adjusted to TL 4** | | | | | **reported Hg concentrations** | | | | |
| --- | --- | --- | --- | --- | --- | --- | --- | --- | --- | --- | --- |
|  |  | mean | SD | median | min | max | mean | SD | median | min | max |
| **all species** | | | | | | | | | | | |
| 1978 | 4 | 935 | 191 | 896 | 779 | 1169 | 240 | 49.0 | 230 | 200 | 300 |
| 1979 | 110 | 2962 | 1815 | 2455 | 206 | 8572 | 760 | 466 | 630 | 53.0 | 2200 |
| 1980 | 70 | 3301 | 1385 | 3117 | 1040 | 8244 | 847 | 356 | 800 | 267 | 2116 |
| ### |  |  |  |  |  |  |  |  |  |  |  |
| 1984 | 131 | 3194 | 1486 | 2850 | 1114 | 9063 | 891 | 494 | 764 | 286 | 2795 |
| 1985 |  |  |  |  |  |  |  |  |  |  |  |
| 1986 | 106 | 2312 | 1332 | 2414 | 341 | 6620 | 697 | 391 | 658 | 118 | 2060 |
| ### |  |  |  |  |  |  |  |  |  |  |  |
| 1991 | 120 | 1758 | 1083 | 1558 | 19.7 | 5844 | 497 | 317 | 445 | 16.0 | 2310 |
| 1992 | 43 | 668 | 712 | 370 | 6.16 | 2455 | 282 | 254 | 220 | 5.00 | 1000 |
| 1993 | 33 | 271 | 297 | 148 | 24.7 | 1405 | 220 | 241 | 120 | 20.0 | 1140 |
| 1994 | 569 | 1206 | 954 | 994 | 12.3 | 10602 | 709 | 588 | 585 | 10.0 | 8600 |
| 1995 | 147 | 795 | 763 | 603 | 49.3 | 5701 | 488 | 369 | 395 | 40.0 | 2300 |
| 1996 | 184 | 757 | 808 | 346 | 6.16 | 4364 | 321 | 256 | 270 | 5.00 | 1470 |
| 1997 | 302 | 1128 | 1296 | 768 | 6.16 | 9258 | 549 | 706 | 400 | 5.00 | 9080 |
| 1998 | 183 | 925 | 1067 | 445 | 74.0 | 8728 | 407 | 302 | 348 | 40.0 | 1390 |
| 1999 | 491 | 1098 | 811 | 935 | 17.3 | 7065 | 563 | 382 | 470 | 14.0 | 3150 |
| 2000 | 500 | 722 | 924 | 392 | 0.0 | 8133 | 255 | 410 | 170 | 0.00 | 7976 |
| 2001 | 353 | 652 | 759 | 386 | 6.16 | 6442 | 235 | 196 | 190 | 5.00 | 1028 |
| 2002 | 396 | 764 | 852 | 448 | 0.00 | 6530 | 302 | 266 | 216 | 0.00 | 1847 |
| 2003 | 297 | 939 | 954 | 642 | 15.7 | 6234 | 320 | 279 | 249 | 10.0 | 2666 |
| 2004 | 319 | 718 | 993 | 377 | 19.6 | 9155 | 278 | 254 | 222 | 16.0 | 2369 |
| 2005 | 379 | 674 | 787 | 399 | 1.43 | 5403 | 236 | 245 | 180 | 1.20 | 1870 |
| 2006 | 358 | 817 | 926 | 498 | 1.22 | 6885 | 292 | 248 | 213 | 1.20 | 1750 |
| 2007 | 385 | 687 | 791 | 469 | 0.00 | 9044 | 294 | 281 | 217 | 0.00 | 2348 |
| 2008 | 386 | 753 | 810 | 495 | 27.5 | 5562 | 253 | 237 | 191 | 7.00 | 2070 |
| 2009 | 519 | 748 | 898 | 468 | 8.96 | 8429 | 263 | 231 | 211 | 2.30 | 1617 |
| 2010 | 197 | 980 | 903 | 713 | 12.3 | 4835 | 321 | 253 | 246 | 10.0 | 1460 |
| 2011 | 167 | 816 | 700 | 814 | 47.7 | 5569 | 262 | 215 | 190 | 33.0 | 1300 |
| 2012 | 100 | 770 | 497 | 754 | 46.9 | 2907 | 240 | 159 | 196 | 59.4 | 971 |
| 2013 | 152 | 1347 | 1079 | 1015 | 139 | 5386 | 313 | 218 | 271 | 40.0 | 1321 |
| **bream** | | | | | | | | | | | |
| 1994 | 172 | 1793 | 1022 | 1642 | 335 | 5416 | 460 | 262 | 422 | 86 | 1390 |
| 1995 | 10 | 1185 | 542 | 900 | 791 | 2127 | 304 | 139 | 231 | 203 | 546 |
| 1996 | 19 | 2102 | 308 | 2104 | 1481 | 2533 | 539 | 79 | 540 | 380 | 650 |
| 1997 | 76 | 1686 | 717 | 1644 | 125 | 3387 | 433 | 184 | 422 | 32 | 869 |
| 1998 | 29 | 2205 | 608 | 2174 | 1091 | 3362 | 566 | 156 | 558 | 280 | 863 |
| 1999 | 153 | 1538 | 502 | 1520 | 351 | 2844 | 395 | 129 | 390 | 90 | 730 |
| 2000 | 96 | 875 | 708 | 658 | 66.2 | 3210 | 225 | 182 | 169 | 17 | 824 |
| 2001 | 43 | 997 | 591 | 736 | 159 | 2065 | 256 | 152 | 189 | 40.9 | 530 |
| 2002 | 73 | 1390 | 757 | 1247 | 321 | 3230 | 357 | 194 | 320 | 82.4 | 829 |
| 2003 | 41 | 1561 | 575 | 1714 | 405 | 2455 | 401 | 148 | 440 | 104 | 630 |
| 2004 | 41 | 1040 | 493 | 1091 | 62.3 | 1987 | 267 | 127 | 280 | 16 | 510 |
| 2005 | 63 | 847 | 607 | 857 | 4.68 | 2022 | 217 | 156 | 220 | 1 | 519 |
| 2006 | 49 | 1332 | 725 | 1243 | 217 | 3717 | 342 | 186 | 319 | 56 | 954 |
| 2007 | 51 | 1090 | 585 | 918 | 245 | 2910 | 280 | 150 | 236 | 63 | 747 |
| 2008 | 74 | 824 | 431 | 765 | 74.5 | 1734 | 212 | 111 | 196 | 19 | 445 |
| 2009 | 175 | 717 | 628 | 619 | 8.96 | 2271 | 184 | 161 | 159 | 2.3 | 583 |
| 2010 | 43 | 846 | 362 | 904 | 77.9 | 1769 | 217 | 93 | 232 | 20 | 454 |
| 2011 | 33 | 976 | 330 | 957 | 273 | 1681 | 250 | 85 | 246 | 70 | 432 |
| 2012 | 28 | 929 | 256 | 892 | 531 | 1563 | 238 | 66 | 229 | 136 | 401 |
| 2013 | 34 | 1274 | 758 | 1122 | 257 | 4617 | 327 | 194 | 288 | 66 | 1185 |

Table S9: **Federal states monitoring: Hg** concentrations (µg/kg wet weight) in fish from the **river Elbe (all sampling sites).** Data refer to individual fish. Left side: Hg concentrations normalized to 26% dry mass (DM) and adjusted to trophic level 4.0 (TL 4). Right side: original reported concentrations.

| **year** | **n** | **Hg concentrations normalized to 26% DM and adjusted to TL 4** | | | | | **reported Hg concentrations** | | | | |
| --- | --- | --- | --- | --- | --- | --- | --- | --- | --- | --- | --- |
|  |  | mean | SD | median | min | max | mean | SD | median | min | max |
| **all species** | | | | | | | | | | | |
| 1994 | 569 | 1206 | 954 | 994 | 12.3 | 10602 | 709 | 588 | 585 | 10.0 | 8600 |
| 1995 | 147 | 795 | 763 | 603 | 49.3 | 5701 | 488 | 369 | 395 | 40.0 | 2300 |
| 1996 | 184 | 757 | 808 | 346 | 6.16 | 4364 | 321 | 256 | 270 | 5.00 | 1470 |
| 1997 | 231 | 1036 | 1266 | 597 | 6.16 | 8825 | 460 | 408 | 340 | 5.00 | 3500 |
| 1998 | 183 | 925 | 1067 | 445 | 74.0 | 8728 | 407 | 302 | 348 | 40.0 | 1390 |
| 1999 | 491 | 1098 | 811 | 935 | 17.3 | 7065 | 563 | 382 | 470 | 14.0 | 3150 |
| 2000 | 114 | 1117 | 1205 | 610 | 31.8 | 7453 | 394 | 293 | 361 | 11.0 | 1405 |
| 2001 | 134 | 879 | 958 | 499 | 49.1 | 6442 | 320 | 198 | 280 | 17.0 | 990 |
| 2002 | 166 | 907 | 989 | 427 | 37.0 | 5611 | 342 | 226 | 270 | 30.0 | 1360 |
| 2003 | 100 | 1335 | 1058 | 1202 | 92.0 | 6234 | 426 | 222 | 380 | 140 | 1340 |
| 2004 | 140 | 744 | 851 | 385 | 45.6 | 6096 | 317 | 254 | 260 | 37.0 | 1660 |
| 2005 | 174 | 555 | 525 | 339 | 15.8 | 2284 | 236 | 251 | 185 | 5.60 | 1870 |
| 2006 | 120 | 770 | 740 | 572 | 62.9 | 4754 | 286 | 219 | 215 | 51.0 | 1250 |
| 2007 | 220 | 720 | 649 | 549 | 48.1 | 5251 | 363 | 290 | 274 | 39.0 | 2110 |
| 2008 | 96 | 891 | 799 | 683 | 28.3 | 3810 | 272 | 191 | 220 | 30.0 | 990 |
| 2009 | 285 | 834 | 951 | 573 | 8.96 | 8429 | 251 | 217 | 211 | 2.30 | 1500 |
| 2010 | 100 | 946 | 790 | 764 | 81.2 | 3657 | 318 | 233 | 246 | 62.0 | 1380 |
| 2011 | 122 | 975 | 731 | 890 | 55.1 | 5569 | 312 | 228 | 239 | 54.0 | 1300 |
| 2012 | 100 | 770 | 497 | 754 | 46.9 | 2907 | 240 | 159 | 196 | 59.4 | 971 |
| 2013 | 100 | 1116 | 679 | 985 | 139 | 4617 | 308 | 216 | 257 | 47.0 | 1321 |
| **bream** | | | | | | | | | | | |
| 1994 | 172 | 1793 | 1022 | 1642 | 335 | 5416 | 460 | 262 | 422 | 86.0 | 1390 |
| 1995 | 10 | 1185 | 542 | 900 | 791 | 2127 | 304 | 139 | 231 | 203 | 546 |
| 1996 | 19 | 2102 | 308 | 2104 | 1481 | 2533 | 539 | 79.1 | 540 | 380 | 650 |
| 1997 | 47 | 1681 | 707 | 1647 | 514 | 3387 | 432 | 182 | 423 | 132 | 869 |
| 1998 | 29 | 2205 | 608 | 2174 | 1091 | 3362 | 566 | 156 | 558 | 280 | 863 |
| 1999 | 153 | 1538 | 502 | 1520 | 351 | 2844 | 395 | 129 | 390 | 90.0 | 730 |
| 2000 | 22 | 1905 | 601 | 2020 | 635 | 3210 | 489 | 154 | 519 | 163 | 824 |
| 2001 | 16 | 1610 | 438 | 1773 | 701 | 2065 | 413 | 113 | 455 | 180 | 530 |
| 2002 | 37 | 1867 | 672 | 1948 | 468 | 3230 | 479 | 172 | 500 | 120 | 829 |
| 2003 | 30 | 1794 | 430 | 1929 | 857 | 2455 | 460 | 110 | 495 | 220 | 630 |
| 2004 | 24 | 1320 | 397 | 1344 | 623 | 1987 | 339 | 102 | 345 | 160 | 510 |
| 2005 | 35 | 924 | 636 | 982 | 21.8 | 2022 | 237 | 163 | 252 | 5.60 | 519 |
| 2006 | 19 | 1470 | 276 | 1473 | 1009 | 1886 | 377 | 70.8 | 378 | 259 | 484 |
| 2007 | 35 | 1270 | 580 | 1146 | 330 | 2910 | 326 | 149 | 294 | 84.7 | 747 |
| 2008 | 23 | 949 | 389 | 1091 | 273 | 1520 | 243 | 100 | 280 | 70.0 | 390 |
| 2009 | 157 | 722 | 650 | 627 | 8.96 | 2271 | 185 | 167 | 161 | 2.30 | 583 |
| 2010 | 27 | 985 | 253 | 1005 | 448 | 1371 | 253 | 64.8 | 258 | 115 | 352 |
| 2011 | 23 | 1045 | 270 | 992 | 629 | 1681 | 268 | 69.3 | 255 | 162 | 432 |
| 2012 | 28 | 929 | 256 | 892 | 531 | 1563 | 238 | 65.8 | 229 | 136 | 401 |
| 2013 | 31 | 1354 | 746 | 1169 | 505 | 4617 | 347 | 191 | 300 | 130 | 1185 |

Table S10: **Federal states monitoring: Hg** concentrations (µg/kg wet weight) in fish from the **Elbe sampling site km 13 (Prossen).** Data refer to individual fish. Left side: Hg concentrations normalized to 26% dry mass (DM) and adjusted to trophic level 4.0 (TL 4). Right side: original reported concentrations.

| **year** | **n** | **Hg concentrations normalized to 26% DM and adjusted to TL 4** | | | | | **reported Hg concentrations** | | | | |
| --- | --- | --- | --- | --- | --- | --- | --- | --- | --- | --- | --- |
|  |  | mean | SD | median | min | max | mean | SD | median | min | max |
| **all species** | | | | | | | | | | | |
| 1994 | 11 | 978 | 165 | 1018 | 650 | 1199 | 1055 | 87.7 | 1054 | 928 | 1215 |
| 1995 | 12 | 501 | 152 | 486 | 323 | 784 | 583 | 161 | 539 | 345 | 927 |
| 1996 | 21 | 1200 | 983 | 1347 | 208 | 3246 | 387 | 143 | 360 | 210 | 780 |
| 1997 | 21 | 1953 | 2293 | 1112 | 265 | 7467 | 746 | 385 | 690 | 197 | 1512 |
| 1998 | 20 | 901 | 691 | 706 | 206 | 2622 | 614 | 353 | 420 | 276 | 1390 |
| 1999 | 23 | 1392 | 1091 | 1237 | 223 | 3948 | 567 | 357 | 460 | 230 | 1570 |
| 2000 | 20 | 1173 | 1191 | 621 | 143 | 4939 | 485 | 313 | 394 | 121 | 1405 |
| 2001 | 20 | 1321 | 1504 | 898 | 275 | 6442 | 400 | 173 | 390 | 180 | 930 |
| 2002 | 20 | 1030 | 895 | 880 | 147 | 4156 | 317 | 165 | 255 | 90.0 | 600 |
| 2003 | 20 | 1181 | 832 | 1314 | 142 | 3048 | 392 | 160 | 380 | 160 | 770 |
| 2004 | 20 | 918 | 614 | 1007 | 208 | 2280 | 374 | 163 | 360 | 140 | 740 |
| 2005 | 20 | 1033 | 556 | 1010 | 213 | 2284 | 418 | 321 | 315 | 153 | 1570 |
| 2006 | 20 | 791 | 1014 | 597 | 152 | 4754 | 328 | 354 | 178 | 85.0 | 1250 |
| 2007 | 20 | 639 | 529 | 521 | 100 | 1821 | 267 | 190 | 223 | 104 | 890 |
| 2008 | 20 | 1135 | 918 | 762 | 123 | 3208 | 286 | 223 | 160 | 70.0 | 830 |
| 2009 | 20 | 1018 | 701 | 1003 | 212 | 2458 | 378 | 203 | 316 | 67.0 | 802 |
| 2010 | 24 | 1010 | 619 | 929 | 266 | 2729 | 371 | 225 | 284 | 131 | 908 |
| 2011 | 20 | 908 | 511 | 946 | 194 | 2145 | 232 | 129 | 168 | 109 | 555 |
| 2012 | 20 | 883 | 670 | 803 | 47 | 2907 | 200 | 119 | 175 | 60.7 | 420 |
| 2013 | 20 | 1304 | 638 | 1266 | 141 | 2311 | 298 | 199 | 252 | 47.0 | 707 |
| **bream** | | | | | | | | | | | |
| 1996 | 2 | 2143 | 165 | 2143 | 2026 | 2260 | 550 | 42.4 | 550 | 520 | 580 |
| 1997 | 4 | 1443 | 735 | 1257 | 769 | 2487 | 370 | 189 | 323 | 197 | 638 |
| 1998 | 2 | 2231 | 554 | 2231 | 1839 | 2622 | 573 | 142 | 573 | 472 | 673 |
| 1999 | 2 | 1773 | 82.7 | 1773 | 1714 | 1831 | 455 | 21.2 | 455 | 440 | 470 |
| 2000 | 2 | 2053 | 11.0 | 2053 | 2046 | 2061 | 527 | 2.83 | 527 | 525 | 529 |
| 2001 | 4 | 1636 | 391 | 1812 | 1052 | 1870 | 420 | 100 | 465 | 270 | 480 |
| 2002 | 4 | 1549 | 538 | 1344 | 1169 | 2338 | 398 | 138 | 345 | 300 | 600 |
| 2003 | 5 | 1808 | 291 | 1714 | 1520 | 2221 | 464 | 74.7 | 440 | 390 | 570 |
| 2004 | 5 | 1208 | 334 | 1364 | 623 | 1442 | 310 | 85.7 | 350 | 160 | 370 |
| 2005 | 4 | 1418 | 165 | 1418 | 1301 | 1535 | 364 | 42.4 | 364 | 334 | 394 |
| 2006 | 1 | 1796 |  | 1796 | 1796 | 1796 | 461 |  | 461 | 461 | 461 |
| 2007 | 4 | 733 | 116 | 723 | 605 | 884 | 188 | 29.8 | 185 | 155 | 227 |
| 2008 | 6 | 468 | 152 | 468 | 273 | 662 | 120 | 39.0 | 120 | 70.0 | 170 |
| 2009 |  |  |  |  |  |  |  |  |  |  |  |
| 2010 | 7 | 896 | 197 | 951 | 608 | 1173 | 230 | 50.6 | 244 | 156 | 301 |
| 2011 | 2 | 793 | 231 | 793 | 629 | 957 | 204 | 59.4 | 204 | 162 | 246 |
| 2012 | 3 | 729 | 144 | 714 | 593 | 881 | 187 | 37.1 | 183 | 152 | 226 |
| 2013 | 5 | 1182 | 408 | 988 | 814 | 1845 | 303 | 105 | 254 | 209 | 474 |

Table S11: **Environmental Specimen Bank: PFOS** concentrations (µg/kg wet weight) and temporal trends in bream (*Abramis brama*) sampled under the German Environmental Specimen Program. Data refer to annual pooled samples of approx. 20 fish each. Left side: PFOS concentrations normalized to 26% dry mass and adjusted to trophic level 4.0 (TL 4). Right side: original reported concentrations. Source: Theobald et al. (2011).

| **Water-body** | **sampling site** | **sampling period** | **n** | **PFOS normalized to standard fish of 26% dry mass and TL** | | | | | | **EQS met in 2014** | **reported PFOS concentrations** | | | | |
| --- | --- | --- | --- | --- | --- | --- | --- | --- | --- | --- | --- | --- | --- | --- | --- |
|  |  |  |  | **mean** | **± SD** | **median** | **min** | **max** | **Linear trend** |  | **mean** | **± SD** | **median** | **min** | **max** |
| Lake Belau | Lake Belau | 1997-2013 | 8 | 3.7 | 1.8 | 3.4 | 1.4 | 6.4 | Decrease: -0.3 µg/kg p.a.; p<0.01 | yes^1^ | 0.9 | 0.4 | 0.8 | 0.3 | 1.4 |
| Saar | Güdingen | 1995-2014 | 11 | 55.0 | 21.2 | 55.4 | 21.0 | 99.5 | Decrease: -1.6 µg/kg p.a.; p<0.01 | no | 14.8 | 5.6 | 14.7 | 6.0 | 26.9 |
|  | Rehlingen | 1995-2014 | 11 | 53.5 | 20.6 | 54.3 | 19.3 | 103.5 | Decrease: -0.8 µg/kg p.a.; p=0.05 | no | 14.2 | 6.1 | 13.2 | 4.5 | 29.3 |
| Rhine | Weil | 1995-2011 | 16 | 152.9 | 32.7 | 151.1 | 103 | 234.4 | Increase: +3.5 µg/kg p.a.; p=0.02 | no | 45.4 | 9.9 | 46.3 | 31.4 | 70.3 |
|  | Iffezheim | 1995-2014 | 18 | 147.6 | 44.5 | 143.8 | 83.6 | 236.3 | no | no^2^ | 39.8 | 12.7 | 39.0 | 19.3 | 65.7 |
|  | Koblenz | 1995-2014 | 18 | 146.3 | 35.5 | 146.2 | 67.1 | 208.2 | Decrease: -2.3 µg/kg p.a.; p=0.04 | no | 38.6 | 9.5 | 37.4 | 18.6 | 60.4 |
|  | Bimmen | 1995-2014 | 18 | 152.4 | 51.3 | 144.3 | 71.7 | 245.3 | Decrease: -5.2 µg/kg p.a.; p<0.01 | no | 37.5 | 12.1 | 37.4 | 17.6 | 62.3 |
| Elbe | Prossen | 1995-2014 | 11 | 82.7 | 33.6 | 80.1 | 40.1 | 148.9 | Decrease: : -4.3 µg/kg pa.; p<0.01 | no | 20.2 | 8.7 | 19.8 | 9.4 | 38.0 |
|  | Zehren | 2013-2014 | 2 | 63.0 | 4.4 | 63.0 | 59.9 | 66.1 |  | no | 15.5 | 0.8 | 15.5 | 14.9 | 16.1 |
|  | Barby | 1995-2014 | 11 | 89.4 | 32.3 | 91.6 | 39.2 | 152.7 | Decrease: -3.8 µg/kg p.a.; p<0.01 | no | 22.0 | 8.7 | 23.4 | 9.5 | 38.9 |
|  | Cumlosen | 2013-2014 | 2 | 44.6 | 2.8 | 44.6 | 42.6 | 46.6 |  | no | 11.2 | 0.5 | 11.2 | 10.8 | 11.5 |
|  | Blankenese | 1995-2014 | 11 | 149.9 | 83.5 | 135.3 | 53.5 | 319.8 | Decrease: -10.7 µg/kg pa.; p<0.01 | no | 41.1 | 24.0 | 38.6 | 14.1 | 91.0 |
| Mulde | Dessau | 2013-2014 | 2 | 50.0 | 3.3 | 50.0 | 47.7 | 52.3 |  | no | 12.6 | 1.8 | 12.6 | 11.3 | 13.9 |
| Saale | Wettin | 1995-2014 | 11 | 32.6 | 11.5 | 33.9 | 14.8 | 46.9 | Decrease: -1.3 µg/kg p.a.; p<0.01 | no | 8.8 | 3.1 | 9.4 | 4.2 | 13.9 |
| Danube | Ulm | 2003-2014 | 10 | 43.9 | 18.0 | 39.8 | 20.6 | 80.4 | Decrease: -4.0 µg/kg p.a.; p<0.01 | no | 11.7 | 4.4 | 10.2 | 6.0 | 19.9 |
|  | Kelheim | 2003-2014 | 10 | 48.2 | 18.7 | 50.5 | 14.8 | 85.1 | Decrease: -4.3 µg/kg p.a.; p<0.01 | no | 12.7 | 5.1 | 13.0 | 3.6 | 22.5 |
|  | Jochenstein | 2003-2014 | 10 | 71.9 | 25.5 | 63.5 | 31.7 | 109.2 | Decrease: -4.5 µg/kg p.a.; p=0.02 | no | 21.2 | 8.4 | 19.2 | 9.6 | 33.1 |

n: number of annual pool samples; ^1^ refers to 2013; ^2^ refers to 2011

Table S12: **Federal States Monitoring: PFOS** concentrations (µg/kg wet weight) in fish from **German freshwaters (all sampling sites)**. Data refer to individual fish sampled in Bavaria (2005) and North Rhine-Westphalia (2006-2010). Left side: concentrations adjusted to a standard fish of 26% dry mass (DM) and trophic level 4.0 (TL 4). Right side: original reported concentrations.

| **year** | **n** | **PFOS concentrations normalized to 26% DM and TL 4** | | | | | **reported PFOS concentrations** | | | | |
| --- | --- | --- | --- | --- | --- | --- | --- | --- | --- | --- | --- |
|  |  | mean | ± SD | median | min | max | mean | ± SD | median | min | max |
| **all species** | | | | | | | | | | | |
| 2005 | 16 | 10.7 | 7.2 | 11.1 | 0.4 | 30.1 | 7.2 | 4.3 | 7.5 | 0.2 | 15.8 |
| 2006 | 342 | 1.6 | 0.5 | 1.6 | 0.4 | 3.4 | 48.6 | 114 | 20.0 | 1.0 | 1180 |
| 2007 | 151 | 68.6 | 80.7 | 34.6 | 1.5 | 368 | 35.1 | 37.0 | 22.0 | 2.0 | 203 |
| 2008 | 172 | 379 | 580 | 91.6 | 3.3 | 2827 | 154 | 221 | 39.4 | 0.6 | 804 |
| 2009 | 470 | 71.3 | 169 | 28.6 | 1.0 | 2105 | 34.0 | 61.0 | 15.2 | 0.4 | 534 |
| 2010 | 117 | 195 | 549 | 76.0 | 1.1 | 4852 | 177 | 367 | 85.3 | 1.0 | 2749 |
| **TL 2.0 – 3.2** | | | | | | | | | | | |
| 2005 | 3 | 18.5 | 10.8 | 16.6 | 8.8 | 30.1 | 4.9 | 2.9 | 6.1 | 1.6 | 7.1 |
| 2006 | 120 | 91.6 | 184 | 57.4 | 7.5 | 1913 | 21.0 | 34.6 | 12.5 | 2.0 | 348 |
| 2007 | 40 | 109 | 97.2 | 76.7 | 7.5 | 368 | 25.9 | 25.0 | 17.5 | 2.0 | 102 |
| 2008 | 63 | 327 | 540 | 85.6 | 3.3 | 2827 | 84.2 | 138 | 23.8 | 0.6 | 666 |
| 2009 | 169 | 97.1 | 217 | 34.1 | 3.3 | 2105 | 23.8 | 53.4 | 7.5 | 0.6 | 496 |
| 2010 | 16 | 589 | 1264 | 96.8 | 7.6 | 4852 | 153 | 311 | 23.3 | 2.3 | 1143 |

Table S13: **Federal States Monitoring: PFOS** concentrations (µg/kg wet weight) in fish from the **river Rhine** **(all sampling sites)**. Data refer to individual fish. Left side: Concentrations normalized to trophic level 4.0 (TL 4) and 26% dry mass (DM). Right side: original reported concentrations.

| **year** | **n** | **PFOS concentrations normalized to 26% DM and TL 4** | | | | | **reported PFOS concentrations** | | | | |
| --- | --- | --- | --- | --- | --- | --- | --- | --- | --- | --- | --- |
|  |  | mean | ± SD | median | min | max | mean | ± SD | median | min | max |
| **all species** | | | | | | | | | | | |
| 2006 | 29 | 52.8 | 49.6 | 37.6 | 6.85 | 193 | 25.4 | 18.8 | 20.0 | 6.00 | 71.0 |
| 2007 | 20 | 40.6 | 48.4 | 22.5 | 5.61 | 216 | 22.6 | 15.4 | 16.0 | 6.00 | 59.0 |
| 2008 | 11 | 58.5 | 62.9 | 45.8 | 7.26 | 236 | 18.0 | 18.7 | 13.8 | 4.20 | 71.0 |
| 2009 | 57 | 27.9 | 23.0 | 19.3 | 2.99 | 110 | 18.5 | 14.2 | 15.0 | 2.60 | 61.9 |
| 2010 | 15 | 63.1 | 61.5 | 42.4 | 15.4 | 213 | 38.7 | 18.8 | 36.9 | 13.4 | 72.0 |
| **TL 2.0 – 3.2** | | | | | | | | | | | |
| 2006 | 14 | 82.7 | 55.7 | 67.0 | 30.1 | 193 | 23.9 | 17.3 | 19.0 | 8.00 | 58.0 |
| 2007 | 6 | 83.9 | 73.8 | 64.9 | 19.8 | 216 | 18.3 | 17.2 | 10.5 | 6.00 | 51.0 |
| 2008 | 10 | 63.6 | 63.8 | 47.0 | 13.8 | 236 | 19.2 | 19.2 | 14.2 | 4.20 | 71.0 |
| 2009 | 12 | 50.5 | 31.6 | 45.5 | 17.0 | 110 | 14.6 | 10.2 | 13.8 | 3.10 | 33.0 |
| 2010 | 3 | 157 | 87.6 | 203 | 56.0 | 213 | 47.3 | 26.3 | 61.0 | 17.0 | 64.0 |

Table S14: **Federal States Monitoring: PFOS** concentrations (µg/kg wet weight) in fish from the **Rhine** **sampling sites km 780 - 870**. Data refer to individual fish. Left side: Concentrations normalized to trophic level 4.0 (TL 4) and 26% dry mass (DM). Right side: original reported concentrations.

| **year** | **n** | **PFOS concentrations normalized to 26% DM and TL 4** | | | | | **reported PFOS concentrations** | | | | |
| --- | --- | --- | --- | --- | --- | --- | --- | --- | --- | --- | --- |
|  |  | mean | ± SD | median | min | max | mean | ± SD | median | min | max |
| **all species** | | | | | | | | | | | |
| 2006 | 19 | 41.0 | 28.4 | 30.9 | 6.85 | 96.3 | 24.3 | 18.7 | 20.0 | 6.00 | 71.0 |
| 2007 | 6 | 21.1 | 6.55 | 19.9 | 14.8 | 33.3 | 24.5 | 9.46 | 27.5 | 11.0 | 35.0 |
| 2008 | 6 | 79.1 | 80.4 | 52.0 | 7.26 | 236 | 24.4 | 23.7 | 15.7 | 5.40 | 71.0 |
| 2009 | 24 | 18.8 | 12.9 | 14.5 | 2.99 | 48.2 | 16.1 | 11.4 | 11.9 | 2.60 | 42.0 |
| 2010 | 14 | 53.1 | 49.7 | 40.9 | 15.4 | 213 | 37.1 | 18.4 | 35.7 | 13.4 | 72.0 |
| **TL 2.0 – 3.2** | | | | | | | | | | | |
| 2006 | 7 | 65.8 | 23.9 | 67.7 | 30.1 | 96.3 | 19.3 | 6.68 | 20.0 | 8.00 | 29.0 |
| 2007 |  |  |  |  |  |  |  |  |  |  |  |
| 2008 | 5 | 93.4 | 80.8 | 55.8 | 45.8 | 235.8 | 28.1 | 24.3 | 16.8 | 13.8 | 71.0 |
| 2009 |  |  |  |  |  |  |  |  |  |  |  |
| 2010 | 2 | 134 | 111 | 134 | 56.0 | 212 | 40.5 | 33.2 | 40.5 | 17.0 | 64.0 |
